# Consensus Paper: Cerebellum and Social Cognition

**DOI:** 10.1007/s12311-020-01155-1

**Published:** 2020-07-07

**Authors:** Frank Van Overwalle, Mario Manto, Zaira Cattaneo, Silvia Clausi, Chiara Ferrari, John D. E. Gabrieli, Xavier Guell, Elien Heleven, Michela Lupo, Qianying Ma, Marco Michelutti, Giusy Olivito, Min Pu, Laura C. Rice, Jeremy D. Schmahmann, Libera Siciliano, Arseny A. Sokolov, Catherine J. Stoodley, Kim van Dun, Larry Vandervert, Maria Leggio

**Affiliations:** 1 Department of Psychology and Center for Neuroscience, Vrije Universiteit Brussel, Pleinlaan 2, 1050 Brussels, Belgium; 2 Mediathèque Jean Jacquy, Service de Neurologie, CHU-Charleroi, Charleroi, Belgium; 3 Service des Neurosciences, Université de Mons, Mons, Belgium; 4 University of Milano-Bicocca, 20126 Milan, Italy; 5 IRCCS Mondino Foundation, Pavia, Italy; 6 Ataxia Laboratory, IRCCS Fondazione Santa Lucia, 00179 Rome, Italy; 7 Department of Psychology, Sapienza University of Rome, Rome, Italy; 8 University of Pavia, 27100 Pavia, Italy; 9 McGovern Institute for Brain Research, Massachusetts Institute of Technology, Cambridge, USA; 10 Ataxia Unit, Cognitive Behavioral Neurology Unit, Laboratory for Neuroanatomy and Cerebellar Neurobiology, Department of Neurology, Massachusetts General Hospital, Harvard Medical School, Boston, MA USA; 11 Service de Neurologie & Neuroscape@NeuroTech Platform, Département des Neurosciences Cliniques, Centre Hospitalier Universitaire Vaudois (CHUV), Service de Neurologie Lausanne, Lausanne, Switzerland; 12 Department of Neurosciences, University of Padua, Padua, Italy; 13 Department of Psychology and Department of Neuroscience, American University, Washington, DC USA; 14 Program in Behavioral Neuroscience, Sapienza University of Rome, Rome, Italy; 15 Department of Neurology, University Neurorehabilitation, University Hospital Inselspital, University of Bern, Bern, Switzerland; 16 Wellcome Centre for Human Neuroimaging, Institute of Neurology, University College London (UCL), London, UK; 17 Neuroscape Center, Weill Institute for Neurosciences, Department of Neurology, University of California San Francisco, San Francisco, CA USA; 18 Neurologic Rehabilitation Research, Rehabilitation Research Institute (REVAL), Hasselt University, 3590 Diepenbeek, Belgium; 19 American Nonlinear Systems, 1529 W. Courtland Avenue, Spokane, WA 99205-2608 USA

**Keywords:** Posterior cerebellum, Crus I/II, Social cognition, Social mentalizing, Mind reading, Social mirroring, Body language reading, Social action sequences, Cerebellar stimulation, Innate hand-tool overlap, Stone-tool making

## Abstract

The traditional view on the cerebellum is that it controls motor behavior. Although recent work has revealed that the cerebellum supports also nonmotor functions such as cognition and affect, only during the last 5 years it has become evident that the cerebellum also plays an important social role. This role is evident in social cognition based on interpreting goal-directed actions through the movements of individuals (social “mirroring”) which is very close to its original role in motor learning, as well as in social understanding of other individuals’ mental state, such as their intentions, beliefs, past behaviors, future aspirations, and personality traits (social “mentalizing”). Most of this mentalizing role is supported by the posterior cerebellum (e.g., Crus I and II). The most dominant hypothesis is that the cerebellum assists in learning and understanding social action sequences, and so facilitates social cognition by supporting optimal predictions about imminent or future social interaction and cooperation. This consensus paper brings together experts from different fields to discuss recent efforts in understanding the role of the cerebellum in social cognition, and the understanding of social behaviors and mental states by others, its effect on clinical impairments such as cerebellar ataxia and autism spectrum disorder, and how the cerebellum can become a potential target for noninvasive brain stimulation as a therapeutic intervention. We report on the most recent empirical findings and techniques for understanding and manipulating cerebellar circuits in humans. Cerebellar circuitry appears now as a key structure to elucidate social interactions.

## Introduction and Evolutionary Past

This consensus paper starts with an introduction on the role of the cerebellum in social cognition by Frank Van Overwalle and Mario Manto and also introduces the less-experienced reader into the functional anatomy and computations of the cerebellum with respect to social cognition. This is followed by a discussion on the potential evolutionary role of stone-tool making for the social cerebellum by Larry Vandervert.

### Introduction (Frank Van Overwalle, Mario Manto)

Research on the relationship between the cerebellum and social cognition is very young and, apart from occasional early contributions, began to emerge over the past 5 years. Prior reports on the social role of the cerebellum were often limited to side aspects of affective processing and anecdotally described cerebellar patients having affective deficits. These reports focused on the understanding of affect in facial expressions of others [1] without much attention to higher-level mental states of others. However, a novel collaboration between researchers from the field of social neuroscience (Frank Van Overwalle) and the cerebellum (Peter Marien and Mario Manto) resulted in the discovery of the important social function of the cerebellum [2, 3] which instigated novel research on the potential role of the cerebellum in social cognition. Social cognitive processes encompass social “mentalizing” (or mind reading) which depends on the inferred unobserved mental state of other people as well as social “mirroring” (or body reading) which depends on the observed goal-directed body movement of others. Research on the cerebrum has documented that these two processes recruit distinct cortical areas.

Social mentalizing is an evolutionary younger function that activates associative cortical areas (in particular, a larger part of the so-called default network) responsible for switching one’s perspective to unobservable mental states of another person (e.g., intentions, desires, and beliefs) and encoding this information at a more abstract level in the form of personality traits and autobiographies—which indicate what kind of person someone is (e.g., meta-analyses by [4, 5]). Key cortical areas are the temporo-parietal junction (TPJ) for here-and-now inferences of intentions and beliefs of others, while the medial prefrontal cortex is responsible for abstract and stable person inferences such as personality traits and preferences. Conversely, social mirroring is an evolutionary older function that activates sensorimotor areas responsible for detecting and understanding biological movement of human body parts (e.g., limbs) such a grabbing a cup and automatically understanding its goal—for drinking (e.g., meta-analyses [6, 7]). Important cortical areas are the posterior superior temporal sulcus (pSTS) which detects biological movement, and key mirror areas involving the anterior intraparietal sulcus (aIPS) which connects particular movements within their typical context (i.e., grabbing a cup with a precision grip at the table or with a full hand grip at the dish washer), and finally the premotor cortex (PMC) which identifies its underlying goal (i.e., for drinking vs. cleaning). Although parts of distinct neural circuits, the pSTS and TPJ are key integrators of sensorimotor and verbal supramodal input, respectively, which are partly overlapping with the pSTS being located more inferior to the TPJ, indicating that body and mind reading are often interacting.

The main hypothesis addressed by many researchers in the field is how the role of the cerebellum in learning, automatizing, and fine-tuning sequences of motor behavior has been extended to the social field, involving sequences of social actions and interactions (e.g., [8]). To test this sequencing hypothesis, research methodologies have been developed that go beyond traditional measures of social cognition and their impairments, in order to identify the assumed role of sequences in social actions and action prediction. To illustrate, cerebellar research quickly incorporated one of the key tasks in social mentalizing: the false belief test. This test involves stories with an agent who does not know that an object has been relocated or changed in his or her absence. Consequently, participants have to realize that the agent lacks information about this change, so that he or she holds a “false” belief about the object’s location or feature, which is no longer true and conflicts with reality [9–11]. Distinguishing between (false) beliefs held by others and reality as one sees it, is a social capacity that is only fully developed by the age of four. The hypothesized sequencing role of the cerebellum is quite evident in false belief stories: It makes a great difference whether a person leaves the room before or after another person hides a loved toy, or tells a secret, and so on. Methodological advances have also been introduced in the study of the cerebellum. This includes not only neuroimaging procedures such as functional magnetic resonance imaging (fMRI) to investigate activated areas in the human cerebellum, and how these areas functionally interact with the cerebral cortex using novel methodologies such as resting-state connectivity and dynamic causal modeling (DCM), but also the novel use of noninvasive cerebellar neurostimulation such a transcranial magnetic stimulation (TMS) and transcranial direct current stimulation (tDCS).

Consensus is growing on the important role of the cerebellum in social cognition, but the field is still at its early stages and in full development. Many early findings and insights are emerging, some of which have proven to be replicable, pointing to the beginning of a substantial body of evidence and a better understanding of the social cerebellum. However, some outcomes are preliminary and need to be treated with caution, while some other studies point to ways for improvement and further research. This consensus paper provides the opinions and reports of a group of selected scientists with established or beginning expertise in the emerging field of the cerebellum and social processing. Given that insight in the social function of the cerebellum holds great promise for a better understanding and treatment of a variety of social impairments, this consensus paper is timely. Opinions are presented as a condensed review of existing research in the field, or as short abstracts of novel research findings in the author’s lab or the larger field.

#### Overview of the Contributions

This consensus paper starts with a discussion on the potential evolutionary role of stone-tool making for the social cerebellum by Larry Vandervert.

The following section involves the role of the cerebellum in mind reading. We start this section with the sequencing hypothesis of the social cerebellum put forward by Maria Leggio, which is an extension of the traditional motor view of the cerebellum and has influenced many current studies on the underlying functionality of the cerebellum in social understanding and prediction. The relationship between social cognition and other motor and nonmotor domains in the cerebellum is further elaborated by Xavier Guell, John Gabrieli, and Jeremy Schmahmann. Their impressive analysis and overview of the twofold task and process gradients in the cerebellum provide again evidence for a domain-specific contribution to social cognition by the cerebellum. In their novel meta-analysis, Qianying Ma and Frank Van Overwalle further document that cerebellar Crus II is mainly involved in social mentalizing. Finally, several tests of Leggio’s sequencing hypothesis are reported in novel empirical contributions by Frank Van Overwalle and his colleagues Elien Heleven, Qianying Ma, and Min Pu.

Next, findings on body reading and action understanding are reported. Marco Michelutti and Arseny Sokolov provide an overview of research on nonverbal body movements (e.g., by point-lights or small markers attached to the major joints while the rest of the body is invisible) and symbolic geometric shape animations. Chiara Ferrari and Zaira Cattaneo discuss the causal role of cerebellar regions involved in biological motion perception. Of interest is that they applied TMS at different time points to delineate the timing of the cerebellar processes in different areas.

A further section elaborates on clinical aspects that are related to the cerebellum. This opens up new perspectives in the clinical practice for treating patients with neurodegenerative, psychiatric, and neurodevelopmental disorders. Silvia Clausi, Michela Lupo, and Maria Leggio provide an overview of the clinical implications of the cerebellar role in mentalizing, which could underlie the difficulties in social cognition reported in cerebellar patients as well as in individuals with social impairments such as autism. Findings on the interaction or connectivity within cerebello-cerebral mentalizing networks and their clinical implications are documented by Giusy Olivito, Libera Siciliano, Frank Van Overwalle, and Maria Leggio. This evidence reveals decreased functional activity and connectivity in multiple cerebello-cerebral networks resulting in impairments in both lower-level mirroring and complex high-level mentalization. Next, Laura Rice and Catherine Stoodley focus on the cerebellar contributions to social behaviors in a specific population: individuals with autism. They discuss promising research with animals and humans on cerebellar structural and functional connectivity to elicit the origin and consequences of cerebellar abnormalities in these populations.

The final section on neurostimulation focuses on possible ways to ameliorate social dysfunctions by cerebellar neurostimulation. Kim van Dun and Mario Manto discuss the social cerebellum as promising target of noninvasive neurostimulation in various impairments of social cognition, while Elien Heleven and Frank Van Overwalle provide preliminary evidence from a pilot study on the effect of cerebellar TMS on performance in social sequencing.

We conclude this consensus paper by highlighting a number of robust findings while pointing out some conflicts and issues where evidence is lacking, along with questions for further research.

#### Cerebellar Areas Involved in Social Cognition

In order to fully appreciate the results of the contributions in this consensus paper for the less-informed reader, it is perhaps instructive to conclude this introduction with a brief description of the functional anatomy of the cerebellum with respect to social processing. Afterwards, we also briefly introduce the computations performed by the cerebellum during social processing.

**Fig. 1:**
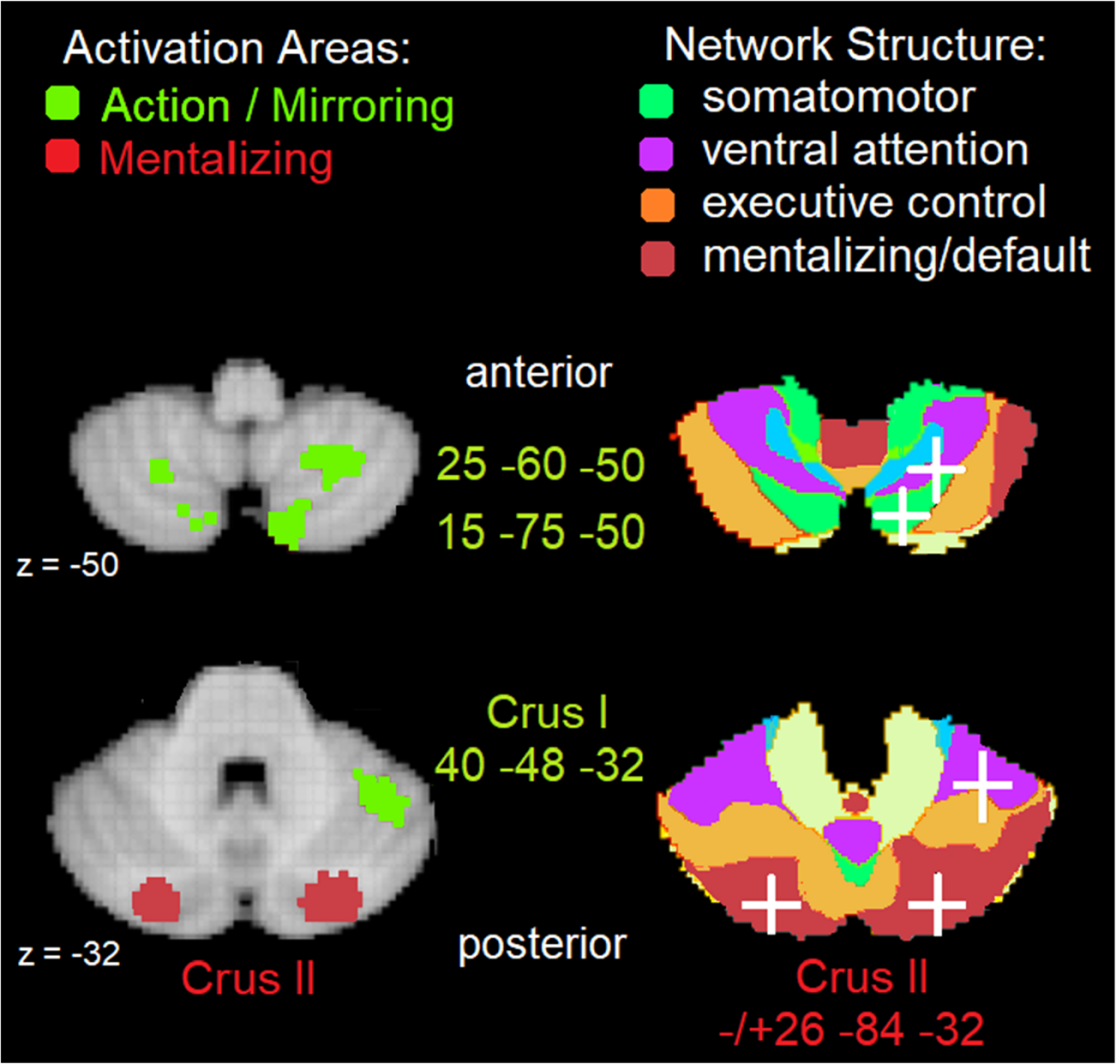
Transversal view of the inferior and superior cerebellum at MNI *z*-coordinates − 50 and – 32, respectively. [Left] The most active areas in the cerebellum from the automated meta-analyses of NeuroSynth (50 topics) [right] overlaid on the 7-network structure of Buckner et al. [12] with coordinates denoted by white crosses. Three green “mirror” areas associated with “action” and “mirror” keywords in NeuroSynth (#19) are part of the green somatomotor integration and purple ventral attention networks; two red “mentalizing” areas associated with the “mentalizing” keyword in NeuroSynth (#8) are part of the red mentalizing network

With respect to functional anatomy, there is a clear preference for motion-related mirroring movement tasks to recruit “somatomotor” networks identified by Buckner and colleagues [12]. In the cerebellum, these are located mainly in the anterior cerebellum parts. For nonmotion-related mentalizing tasks, the “default/mentalizing” network located in the posterior cerebellum is recruited. This can be observed in Fig. 1, where activity given sensorimotor action observation/mirroring versus mental state inferencing was located using NeuroSynth, an internet platform for large-scale, automated synthesis of fMRI data (https://neurosynth.org; see [13]). From this platform, we selected two meta-analyses specified by the keywords “action” and “mirror” on the one hand, and “mentalizing” on the other hand, and then located major areas of activity on top of the 7-network cerebellar structure from Buckner and colleagues [12]. As can be seen (Fig. 1, right panel), “action/mirroring” tasks such as the observation of human hand and arm movements (e.g., [14]) or point-light displays of body movements [15–17] showed activation in areas (denoted by green MNI coordinates) roughly between the anterior − 45 and posterior − 75 *y*-coordinate. Activity is located within the somatosensory integration network as one might expect, but also in the ventral attention network. On the other hand, “mentalizing” tasks including animations of geometric shapes moving in a human-like fashion [18] as well as more high-level mental states, beliefs, and personality traits of others [2] showed activation in areas (denoted by red MNI coordinates) at the posterior − 84 *y*-coordinate. This activity is overwhelmingly located in the mentalizing/default network [12].

This NeuroSynth analysis in Fig. 1 is largely in line with the meta-analysis on the social cerebellum by Van Overwalle et al. ([2]; see Table 1), and their functional interpretation in terms of the 7-network structure by Buckner and colleagues [12]. Mirroring tasks largely recruit the somatomotor network, but Table 1 shows also robust left-hemispheric clusters (which were relatively small in NeuroSynth), and again evidence for a cluster in the ventral attention network. Mentalizing tasks confirm predominant activity in the default/mentalizing network beyond the posterior − 84 *y*-coordinate, but also in the anterior lobule IX. Table 1 provides additional information on mentalizing about the self, which activates the anterior lobules IV and VI at the border of the limbic and somatosensory networks, presumably reflecting proprioceptive and emotionally triggered experiences.

**Table 1:** Meta-analysis by Van Overwalle et al. [2] and interpretation by the 7-networks by Buckner et al. [12]

Tasks	Cerebellar label	Label	Volume	*x*	*y*	*z*	Network (Buckner et al. [12])
Mirroring	Right posterior—uvula	VIIB	800	9	− 78	− 43	Somatomotor integration
	Right anterior—culmen	VI	504	38	− 54	− 29	Ventral attention
	Left posterior—uvula	VIIB	360	− 13	− 78	− 32	Somatomotor integration
	Left posterior—inf. semi-lunar	VIIB	232	− 23	− 68	− 49	somatomotor integration
Event mentalizing	Left posterior—uvula	Crus I	4128	− 24	− 87	− 33	Mentalizing—default
	Right posterior—pyramis	Crus I	624	19	− 81	− 38	Mentalizing—default
Person mentalizing	Right posterior—tuber	Crus I	2544	23	− 82	− 38	Mentalizing—default
	Right anterior—lingual (self^a^)	IV	1016	8	− 45	− 26	Limbic and somatomotor
	Right anterior—culmen (self^a^)	VI	576	27	− 39	− 24	Limbic and somatomotor
Abstraction in mentalizing	Right posterior—uvula	Crus I and IV	8112	15	− 85	− 36	Mentalizing—default
	Right posterior—tonsil	IX	4064	8	− 46	− 49	Mentalizing—default
	Left posterior—tuber	Crus I	816	− 12	− 83	− 38	Mentalizing—default

Note: Anatomical labels given according to the atlas of ALE and Schmahmann et al. [19]. Volume in mm^3^ for each cluster > 200 mm^3^; *x*–*y*–*z*-coordinates converted to MNI

^a^Cluster preferentially involved in self-references

#### Cerebellar Computations Involved in Social Cognition

One of the most adaptive functions of the brain is to predict upcoming sensory, motor, and cognitive states and to correct errors in these predictions in order to avoid repeating them in the future. In this process, the cerebellum has been proposed as having the central function of generating internal models, which are internal representations of the environment, agents, and events, including predictions on future consequences based on these representations. According to the sequencing hypothesis advanced by Leggio and Molinari ([8]; see also [20]; and contribution by Maria Leggio on “The sequencing hypothesis of the social cerebellum”), the major function of these internal models is learning and representing repetitive patterns of temporally structured events, or sequences. This function is captured by *forward* internal models [21], that not only represent how sequences of events unfold over the course of time, but also predict their consequences, such as the effect on the external environment as well as the effect on one’s own proprioceptive experiences [22]. These sequence predictions are based on information received from the cerebral cortex (i.e., *efference copies*), such as social inferences on the other person’s movements received from the pSTS in the cortical mirror network [16], or the other person’s mental state received from the TPJ in the cortical mentalizing network [23, 24]. When predictions do not match, forward models send out exteroceptive and proprioceptive prediction errors to the cortex [22]. This allows the cortex to gradually minimize future errors when repeating the action, observation, or cognitive process. When errors reoccur repeatedly, this might allow the cerebellum to adapt existing internal models to systematic changes in the environment, or develop new models for distinct circumstances [25]. This view on decreasing prediction errors and adaptation of existing models is in line with predictive coding [26–28] and supervised (i.e., error-correcting) connectionist models of neural functioning [25, 29] that have also been applied to social cognition ([30, 31]; for a review [32]). In social cognition, for example, when a sequence of actions performed by another person suggests a false belief, we become immediately aware of potential mistakes that the other person might take. If further actions do not confirm this prediction and suggest that the person was correct after all, error predictions immediately signal us to adjust our false belief inferences and predictions about the person’s future actions.

The circular communication and adjustments of internal models are accomplished through a series of parallel closed-loops from the cortex to the cerebellum, and back. The uniformity of the cerebellar architecture and physiology suggests similar or “universal” computations on incoming signals from the cortex [33], but also suggests functional domain specificity dictated by the distinct areas where these closed-loops terminate in the cerebellum ([21, 34]; see also the contribution by Xavier Guell, John Gabrieli, and Jeremy Schmahmann on “Relationship between cerebellar social cognition and other motor and non-motor domains”). However, it is also possible that the uniform cerebellar circuitry performs a set of multiple computations and functions that are more or less diverse, rather than universal, driven by the input from the distinct cortical inputs [34], and that evolved in parallel with the evolutionary expansion of the cerebellum and cerebral cortex.

Apart from sequences per se, timing of event sequences in the realm of milliseconds up to a second is also a very crucial function of the cerebellum in producing and understanding observed events [25, 35]. In the social domain, timing is crucial in action observation, coordination, and interaction, as exemplified by the difficulty experienced in social interaction using virtual platforms when experiencing small transmission delays in voice and vision. However, it is unclear to what extent millisecond timing is important in mentalizing, because this cognitive process operates at a relatively high abstraction level, largely devoid of rapid feedback from the environment. This is an interesting issue for further research.

A distinct category of internal models generates motor commands and predictions on one’s own action sequences in the pursuit of one’s desired (social) goal and is termed internal *inverse* models [22]. The term inverse reflects reasoning backward from goal to action, to infer to the required action steps. These inverse action models are critical in planning and coordinating the actions required for efficient social interaction and cooperation. For instance, they compute the intended position or expression of the body (e.g., gaze) as input and estimate the motor commands needed to transform the current position or expression into the desired one; or they compute the intended social role of the self in a group (e.g., taking leadership in the center) and estimate the required actions to move from the current role to the desired one.

### The Cerebellum-Driven Social Learning of Inner Speech in the Evolution of Stone-Tool Making and Language: Innate Hand-Tool Connections in the Cerebro-Cerebellar System (Larry Vandervert)

Vandervert [36, 37] argued that due to the required (1) repetitiveness and (2) social learning of the actions of others, stone-tool and language evolution was predominantly cerebellum-driven. He proposed that this repetitive social learning occurred within the framework of theory of mind (ToM) (one’s simulative capacity to make inferences about the mental states of others) [20, 38–40]. Vandervert based this argument on anthropologist Dietrich Stout’s and neuro-anthropologist Erin Hecht’s [41] detailed analysis of the rigorous skill development necessary in learning stone-tool making from others. Their rather detailed findings included, in part, the following critical aspects of social and cognitive skill development required of the learner:

The key bottleneck in the social reproduction of knapping is thus the *extended practice* [italics added] required to achieve perceptual-motor competence. This requires mastery of relationships, for example between the force and location of the strike and the morphology, positioning, and support of the core [42–44], that are not perceptually available to naïve observers and cannot be directly communicated as semantic knowledge. (p. 7862)

Vandervert [36, 37] pointed out that, in their overall description of the evolution of stone-tool knapping and the brain, Stout and Hecht [41] failed to mention (1) the role of the cerebellum in socially mediated skill development [39] and (2) the role of cerebellum-driven inner or silent speech in working memory as found by Marvel and Desmond [45] and Marvel, Morgan, and Kronemer [46].

#### The Evolutionary Emergence of Theory of Mind Through Inner Speech

Stout and Hecht’s [41] above descriptive quote seems to involve mostly observational learning and little or no verbal, goal-related information for success in the social reproduction of knapping. Therefore, some may question whether mentalizing (i.e., ToM) is actually involved in learning stone knapping, as Vandervert [37] suggests. In this regard, Van Overwalle and Baetens [7] discussed the differentiation between the mirror and mentalizing systems in the brain, with mentalizing involving, in part, goal-related verbal information. Accordingly, the position in this article follows Vandervert’s [37] evolutionary approach to ToM, where he proposed that the evolution of stone-tool making gradually produced ToM capability from pre-speech subvocalizations. Vandervert proposed that this pre-speech subvocalization was associated with evolutionarily early mentalizing and that the selection advantages of cerebellar prediction, error-correction, and automaticity of this highly social process led to the *phonological loop* in working memory and to language. That is, language evolved not primarily from communication but primarily from inner speech associated with the learner’s construction of ToM pertinent to the rigors and social context of stone-tool making skills of the teacher. Vandervert argued that this phonological loop-to-language evolution was driven by newly intricate patterns of goal-related attention to increasingly more subtle and repetitive cause-and-effect knapping requirements, and that this was mediated primarily over the last one million years [47–49] by an emerging social–cognitive cerebellum. Thus, while overt semantic teaching may not be helpful in acquiring/teaching stone-tool knapping, Vandervert proposed subvocal speech (self-talk) and therefore early ToM is key, along with perceptual motor learning, to learning/teaching those knapping skills.

The key elements of Vandervert’s [37] foregoing approach to the social origins of ToM are supported specifically by the following studies associated the stone-tool making–inner speech–social cerebellum origins of verbal working memory.

1. Phonological encoding led to evolutionary acquisition of verbal working memory [50].
2. Inner speech increases with task demand [51].
3. Private inner speech in the young learner increases with task demand [52].
4. The key difference between chimpanzee and human learning is that humans have a greater propensity to pay attention to their own and others’ (social) action details [53].
5. Specific cerebellar-posterior parietal processing occurs as verbal information enters phonological storage [45, 54].

The purpose of this article is to offer further support for a cerebellum-driven social learning explanation of the evolution of stone-tool making. This additional support is based on the findings of the existence of (1) an innate hand-tool overlap in the cerebrum [55] and (2) specific tool modules in the lateral posterior cerebellum for both actual and imagined tool use [56, 57].

#### An Innate Hand-Tool Overlap in the Cerebrum and Tool Modules in the Lateral, Posterior Cerebellum

At least two important lines of evidence support Vandervert’s [36, 37] contention. *First*, Higuchi, Imamizu, and Kawato [56] and Imamizu and Kawato [57] found that both the *actual* and *imagined* use of tools are modularized in the cerebellum (with specific modules for scissors, hammer, screw driver, and so forth). These modularized models of tool use (especially the imagined use of tools) are found largely in the newly evolved lateral cerebellar hemispheres which have expanded greatly over the last one million years. The cerebellum’s dentate nucleus sends both actual tool use and imaginary tool use models to the cerebral cortex where they can be consciously experienced [58]*. Second*, in studying dysplasics (individuals born without hands), Striem-Amit, Vannuscope, and Caramazza [55] have described the evolution of an *innate* hand-tool overlap area in the occipital–temporal area of the cerebral cortex for the acceptance of tools into the hand:

The hand tool overlap would have emerged because of the potential advantage that accrues from the efficient processing of hands and tools as parts of a common (or closely intertwined), specialized system [tools being advantageous ancillaries. This system, in turn, is connected to the dorsal, action-processing areas [parietal cortex] to allow quick and efficient shaping of hands to grasp and use tools [requiring both phylogenetic and ontogenetic cerebellar refinement]. Once evolved, this innately determined system would manifest itself ontogenetically even in the absence of any of the specific inputs, as in the case of the dysplasics, that originally contributed to the full usefulness of the pattern. (p. 4790)

Although Striem-Amit, Vannuscope, and Caramazza do not specifically mention stone-tool evolution as giving rise to the hand-tool overlap, it is suggested that this innate hand-tool overlap evolved in the brain over at least the last million years [47, 49, 59] of progressively refined stone-tool making and stone-tool use and the expansion of the social–cognitive cerebellum.

#### Combining the Innate Hand-Tool Overlap with Tool Modularization in the Lateral Cerebellum

Following Van Overwalle, Van de Steen, and Mariën [23], it is suggested that a closed-loop, social mentalizing connection between the temporo-parietal junction area of the cerebral cortex (Striem-Amit, Vannuscope, and Caramazza’s [55] hand-tool overlap area) and the lateral posterior cerebellum ([56, 57] tool modules) would jointly optimize social cognition for tool making within ToM construction [36, 37]. It is further suggested that within this social mentalizing during tool making, the cerebellum would be predominant in optimizing the shape of the hand to grasp and the dynamics of its grasp [60].

#### Conclusion

In parallel with the evolution of the cerebellum-driven refinement of inner speech–mediated production of ToM, tools became *embedded* along with the hand in the area specializations of the innate hand-tool overlap [55] and in the cerebellum’s actual and imagined tool modular representations [56, 57]. Moreover, since the cerebellum apparently is key to the refinement of the dynamics of grasp [60], and since, according to Stout and Hecht’s [41] analysis at the beginning of this article, that refinement is socially driven, the evolution of tools was largely a product of the evolving social cerebellum as described by Van Overwalle, Manto, Leggio, and Delgado-Garcia [20]. Vandervert [36] proposed that this story of the social cerebellum was largely the story of the rise of *Homo sapiens*.

## The Cerebellum and Mind Reading

This section starts with the sequencing hypothesis of the social cerebellum put forward by Maria Leggio, which is an extension of the traditional motor view of the cerebellum. Xavier Guell, John Gabrieli, and Jeremy Schmahmann elaborate on the relationship between social cognition and other motor and nonmotor domains in the cerebellum and provide further evidence for a domain-specific contribution to social cognition by the cerebellum. Qianying Ma and Frank Van Overwalle present a novel meta-analysis which documents that cerebellar Crus II is mainly involved in social mentalizing. Finally, several tests of Leggio’s sequencing hypothesis are reported in novel empirical contributions by Frank Van Overwalle and his colleagues Elien Heleven, Qianying Ma, and Min Pu.

### The Sequencing Hypothesis of the Social Cerebellum (Maria Leggio)

A fundamental component of social cognition is the capacity to estimate the mental states of others [61, 62]. Having a sense of another individual’s state of mind requires the creation of a mental model of that individual and the ability to predict how their mental states might influence their behaviors [21]. This process also allows us to recognize when the outcome of a social interaction deviates from our expectations and to use this information to calibrate future social predictions [21].

In complex mentalizing processes, predictions are made possible by stored internal models of human behaviors based on the expectation that actions will be efficient and consistent with individual beliefs, personality traits, and social norms [62]. It has been suggested that the cerebellum plays a role in predictive processing, acting as a forward controller [20, 21, 63], and sequence detection could be its operational mode [8, 64, 65]. Indeed, according to the “sequence detection theory,” the cerebellum detects and simulates repetitive patterns of temporally or spatially structured events, regardless of whether they constitute the sensory consequences of one’s actions in motor planning, expected sensory stimuli in perceptual prediction, or inferences of higher-order processes (e.g., cognitive processes) [8, 64, 65]. This simulation allows internal models to be created [66], and these internal models can be used to make predictions about future events that involve any type of component, such as the body, other persons, or the environment.

At a more complex conceptual level, it has been proposed that the cerebellum is involved in the construction of internal models of mental processes during social interactions, in which the prediction of sequential events plays a central role [20, 38]. In fact, social mentalizing, the more reflective and conscious component of social cognition, has the capacity to attribute mental states to others and adopt the perspective of the other person to make predictions about imminent or future social behavior [67, 68]. Thus, analogous with information processing in the sensorimotor domain, the cerebellum might modulate higher-order cortical activity [23, 69, 70] by detecting socially predictable sequences (e.g., internal model of a social action) and promoting the optimized feedforward control that is necessary to accomplish these functions in a fluid and automated manner [20, 21, 38]. In this way, two main requirements of social interactions can be accomplished: to understand and anticipate actions by one’s self and other persons and to understand the consequences for the self and to recognize deviations in the predicted outcomes of social interactions to modify future social expectations [20, 38].

In a recent fMRI study in healthy subjects, Heleven and colleagues [71] showed that constructing social sequences of actions which require understanding the mental state of the protagonist (e.g., involving false or true beliefs) strongly activates the posterior cerebellum, mainly Crus I–II, which is implicated in more complex and abstract aspects of social cognition [21, 38]. These data are in line with evidence showing cerebellar activation when social predictions are violated (e.g., violations of social norms) [72].

Interestingly, Clausi and colleagues [38] found that patients with degenerative cerebellar atrophy were impaired not only in lower-level and automatic processes of others’ mental state estimation (e.g., body reading) but also in the more complex conceptual level of mentalization, as evidenced by affected performances in the advanced ToM task [73] and in social “faux pas” stories [74]. In these tasks, sequential events are unexpected and ambiguous (e.g., when it is required to accurately identify the underlying intention behind a character’s utterance that is not literally true or to understand that a speaker says something without considering that the listener might not want to hear it or might be hurt by what has been said), requiring constant comparison between the event and the social expectation and a high level of prediction [38]. Otherwise, when the patterns in the stories required a minor level of prediction and error monitoring, such as in “no-faux pas” stories and in the Emotion Attribution test [75], cerebellar patients showed good performance [38].

To provide further support for cerebellar specificity in generating appropriate social action sequences, Van Overwalle and colleagues [3] described impaired abilities in patients affected by cerebellar degenerative disease when performing a sequential version of a false belief task [76]. Taking into account this evidence, it can be conceptualized that the cerebellum is a unique predictive structure in different domains. Like with sensorimotor control, in social cognition, the cerebellum may act by matching external information (social inputs) with the internal model of a specific social event linked to previous experiences, contributing to forming judgments about the mental state of others. Consequently, when there is cerebellar damage, the required fast and continuous exchange of information between the external stimuli and the internal model might be affected, thus interfering with the capacity of the cerebellum to recognize deviations/errors in the outcome of a social interaction and with it its ability to use this information to regulate and adjust future social expectations [38].

In line with this theory, structural and functional alterations within cerebello-cortical networks that are involved in different aspects of social interactions have been described in patients affected by cerebellar damage [21, 38, 77–79]. Further details are reported in the contribution on “Connectivity within the cerebello-cerebral mentalizing network and clinical implications” by Olivito et al.

Impaired sequencing and prediction mechanisms are thought to affect social abilities in several neuropsychiatric and neurodevelopmental pathologies characterized by cerebello-cerebral dysfunctions [20, 21, 38, 77, 79]. Within this framework, to give a few examples, in schizophrenia, alterations in forward modeling are considered to be the cause of hallucinations because of the inability to distinguish between internal states and external events [80, 81]. In autism spectrum disorders, the main behavioral hallmark is an impairment in the ability to recognize and attribute mental states to others to explain and predict their behaviors [82]. A comparison between mentalizing abilities of cerebellar patients and autistic subjects is reported in the contribution on “Clinical Implications of the cerebellar role in mentalizing” by Clausi et al.

Overall, the typical role of the cerebellum in adaptive control and predictive coding in the sensorimotor domain needs to be extended to the social cognition domain because anticipation, adaptation, and learning appear to be indispensable for successful social interactions and adaptive social behavior.

### Relationship Between Cerebellar Social Cognition and Other Motor and Nonmotor Domains: Insights from Human Functional MRI (Xavier Guell, John D.E. Gabrieli, Jeremy D. Schmahmann)

Large fMRI databases such as the Human Connectome Project (HCP) [83] (*n* = 1003) have made it possible to analyze in vivo human cerebellar organization with unprecedented power. Our analyses of cerebellar task and resting-state HCP data have identified a triple representation of nonmotor task activation in the cerebellum [84], described functional gradients in the cerebellar cortex [85], and characterized cerebellar task-based functional topography in the largest dataset analyzed to date [84]. Social task in HCP contrasted a *mentalizing* (theory of mind) condition where participants viewed socially interacting moving geometric objects minus a *random* condition showing randomly moving geometric objects [86, 87]. HCP participants also completed resting-state, motor, working memory, emotion, and language fMRI tasks. Here we analyze social-related processes with respect to other motor and nonmotor domains in the cerebellar cortex as indexed by HCP, specifically through the lens of task activation topography, the principle of multiple representations, and functional gradients.

#### Task Activation Analyses

**Fig. 2:**
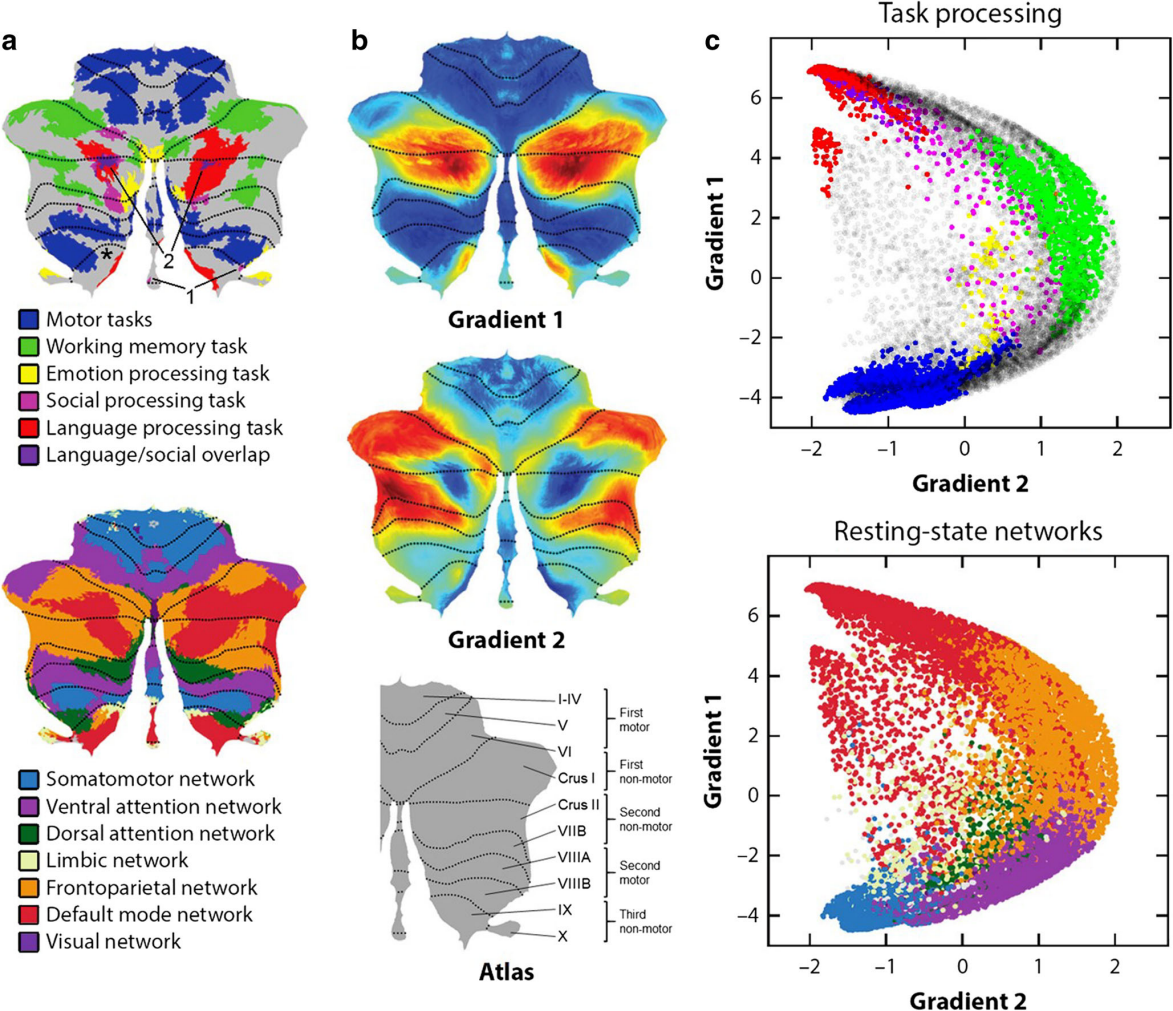
**a** Cerebellar task activation maps [84] (top) and resting-state networks [12] (bottom). 1 = indication of emotion processing activation in lobule IX (for clarity). 2 = indication of area of language/social overlap (for clarity). Asterisk (left lobule IX) = indication of region of working memory task activation if a lower effect size threshold is used, as shown in the supplementary material of [84]. **b** Cerebellar functional gradients [85]. Atlas indicates the position of each motor and nonmotor representation [84]. **c** Relationship of functional gradients 1 and 2 with task activation maps (top) and resting-state networks (bottom). Each dot corresponds to one cerebellar voxel; vertical/horizontal position of each dot corresponds to gradient 1/gradient 2 values for that voxel; the color of each dot indicates whether each voxel belongs to a particular task activation (top) or resting-state network (bottom) map [85]

Task activation maps showing overlap between the territories of social cognition and other functional domains were consistent with existing views [88] that there is a cerebellar domain-specific contribution to social cognition. Social task engaged predominantly lobules Crus I and II as well as lobule IX (Fig. 2a; arrow “1” points at IX activation). Medium effect size thresholds revealed no overlap between social and motor, working memory, or emotion tasks (Fig. 2a) [84]. Social activation overlapped with language activation (Fig. 2a, see arrow “2”), but this overlap was likely due to psychological commonalities between the two tasks. Specifically, language task in HCP contrasted a *Story* condition where participants listened to stories minus a *Math* condition where participants answered arithmetic questions. There was thus a mentalizing component in HCP’s assessment of language processing. In addition, attentional task-focused demands were subtracted by using *Math* as a control condition. As a result, cerebellar language activation largely resembled maps of task-unfocused, default mode processing (see language vs. default mode in Fig. 2a). An overlap between social and default mode processing in the cerebellar cortex has been described previously [88], and this overlap is consistent with a large body of evidence supporting a default network role in social cognition–related processes, in particular social mentalizing [89, 90]. A domain-specific cerebellar contribution to social cognition is inferred from a lack of overlap between social and nonsocial task activation in the cerebellum [84, 88].

#### General Organizational Principles

A different line of inquiry examined general organizational principles that are shared between social and other motor and nonmotor domains. Following well-established descriptions of a double motor representation in lobules I–VI and VIII [91], our analyses indicated that there are also multiple representations of nonmotor task processes in the cerebellar cortex. Specifically, all nonmotor processes in the cerebellar cortex might engage, simultaneously, some aspects of lobules VI/Crus I (first nonmotor representation), lobules Crus II/VIIB (second nonmotor representation), and lobules IX/X (third nonmotor representation) [12, 84] (see atlas in Fig. 2 for an indication of the position of each representation). Of note, first and second nonmotor representations can be contiguous (as in language task or default network in Fig. 2a) or separate (as in working memory task or frontoparietal network in Fig. 2a). Social processing in HCP exhibited a first and contiguous second representation in lobules Crus I/II and a third representation in lobule IX (see arrow indicated by “1” in Fig. 2a, pointing at IX activation) [84]. Cerebellar social neuroanatomy is thus contextualized within a larger triple-representation principle that is common across numerous (possibly all) nonmotor domains in the cerebellar cortex.

#### Functional Gradients in the Cerebellum

Additional insights into the anatomical and psychological architecture of cerebellar social cognition can be obtained from mapping social task activation with respect to cerebellar functional gradients [85] (Fig. 2b). These gradients define the position of, and relationship between, functional territories in the cerebellar cortex. Gradient 1 explained the highest amount of variability in functional connectivity patterns and extended from motor to default mode processing territories (Fig. 2c). Gradient 2 isolated task-focused attentional/executive processing. HCP social task spanned across a wide range of gradient 1 values, with some preference toward high gradient 1 (default mode) territories (see pink and purple color in Fig. 2c, top). This widespread location was in clear contrast with other HCP tasks such as language task (story listening minus math; red in Fig. 2c, top) that was located predominantly at high gradient 1 values, motor task located predominantly at low gradient 1 values (blue in Fig. 2c, top), and working memory task located at high gradient 2 values (green in Fig. 2c, top). The wide distribution of social task activation along functional gradient space resonates with a multimodal understanding of social processing in the cerebellum, engaging multiple levels of information processing along the principal dimensions of cerebellar functional neuroanatomy, with no exclusive localization at any of its poles (default mode, attentional/executive, or motor). In this way, these data illustrate that cerebellar social cognition may engage multiple modalities of brain function including sensorimotor, attentive, inattentive, externally oriented, and internally oriented thought. A similar central location along functional gradients was observed for emotion processing (yellow color in Fig. 2c, top); emotion processing did not conform to a purely default mode (high gradient 1 values), motor (low gradient 1 values), and attentional/executive (high gradient 2 values) division.

#### Implications for Social Processing of Multiple Representations in the Cerebellum

The significance of multiple representations of social processing in the cerebellum (first and contiguous second representation in Crus I/II, third representation in IX) might be elucidated by comparing social processing to other motor and nonmotor domains in cerebellar functional neuroanatomy. The position of each territory of motor and nonmotor representation along cerebellar functional gradients 1 and 2 indicates that functional differences may exist not only between the two motor but also between the three nonmotor representations (see third figure in [85]). Further, because second motor and third nonmotor representations are both located in more central positions along functional gradients 1 and 2 when compared to first motor and first/second nonmotor representation territories, it is possible that second motor representation shares functional similarities with third nonmotor representation (as discussed in [85]). In this way, insights into the role of cerebellar lobule VIII in motor processing (compared to motor processing in lobules I–VI) might provide insights into the role of cerebellar lobule IX in social processing (compared to social processing in Crus I/II).

#### Social Cognition as an Exemplar of the Dysmetria of Thought Theory

The neuroimaging findings presented here, and their significance for the relationship between social cognition and other motor and nonmotor domains, are strongly connected to the universal cerebellar transform (UCT) and dysmetria of thought theories [1, 33, 92–95]. The UCT hypothesis states that all cerebellar contributions to behavior are supported by a singular neurological computation. This theory is predicated on two contrasting and complimentary realities: the paracrystalline repeating cytoarchitecture of the cerebellar cortex [96], set against the topographically precise map of anatomical connections linking distinct regions of the cerebellar hemispheres and nuclei to different cerebral motor, cognitive, and affective areas [33, 92, 97–103]. The uniform structure of the cerebellar cortex enables a unique computation, the UCT, that modulates multiple streams of information processing in the cerebral hemispheres, including social cognition. The dysmetria of thought theory is a corollary of the UCT hypothesis. Because all cerebellar contributions to behavior emerge from a uniform neurological computation, neurological symptoms and signs that are a consequence of cerebellar damage reflect a common neurological dysfunction, namely, dysmetria. In the motor domain, cerebellar lesions result in dysmetria of movement and degrade the coordination, precision, and fluidity of motor control. In the cognitive and affective domains, cerebellar malfunction leads to dysmetria of thought and impairs the coordination, precision, and fluidity of thought and emotion, including social processing [33, 92, 93, 103].

The analysis of functional gradients and their relation to task activation maps follows from the understanding that behaviors are emergent properties of distributed neural circuits linking multiple unique nodes geographically distributed through the nervous system. This is as true for the circuitry subserving motor control [104] and attention [105] as it is for the complex social and moral reasoning Fox [106] required for social processing, and as true for the cerebellum as it is for cerebral cortical areas, thalamic nuclei, and sectors of the basal ganglia [107]. For example, cerebellar lobules V and VIII, while physically distant, are both located close to the motor pole of the principal gradient of cerebellar functional organization. An independent relationship between the spatial location of cerebellar task activation maps and their distribution along cerebellar functional gradients can also be observed in social cognition. The distribution of social task activation maps along a broad spectrum of functional gradient values indicates that cerebellar social processing engages multiple modalities of brain function, such as externally and internally oriented thought. This observation is independent of and compatible with the fact that there are different regions in the cerebellar cortex that are specifically engaged in processes relevant for social cognition. Neural circuits subserving social cognition recruit more than one cerebral cortical area, and more than one cerebellar area with which those cerebral areas are interconnected. The distributed neural circuit therefore exists within the cerebellum itself, a consequence of both the cerebro-cerebellar linkage and of the first, second, and third representations of cognition within the cerebellum. These considerations are consistent with the anatomical principles that guide the formulations of the UCT theory, and with the dysmetria of thought theory, a proposed theoretical underpinning of the cerebellar role in social cognition [108, 109].

### The Domain-Specific Role of the Posterior Crus II in Social Mentalizing (Qianying Ma, Frank Van Overwalle)

Accumulating evidence suggests that the cerebellum supports social cognition [2, 88]. Arguably, the most advanced human social cognitive function involves interpreting another person’s mind, termed *mentalizing* [5, 7, 110]. It requires insight in the mental state of another person or the self, ranging from understanding concrete here-and-now intentions, causes, emotions, and beliefs, to abstract and distant social inferences in terms of personality traits and past, future, or hypothetical events [111–115]. Social mentalizing is subserved by the posterior cerebellum [2, 88], which is evolutionary younger [116], and in particular, by the mentalizing/default network of the cerebellum [12].

However, the question remains to what extent the posterior cerebellum is preferentially engaged by mentalizing, and if so, which areas of the posterior cerebellum? Past functional meta-analyses of the cerebellum did not report social processes but rather classic functions of motor perception and nonmotor cognitive functions such as semantics, language, and executive control [117, 118] or reported a very limited range of social tasks such as biological motion perception of geometric shapes, which is not very representative of human social mentalizing [84]. Even the most extensive meta-analysis to date by Van Overwalle et al. [2] did not weigh the importance of social functions in the cerebellum in comparison to other nonsocial processes.

**Fig. 3:**
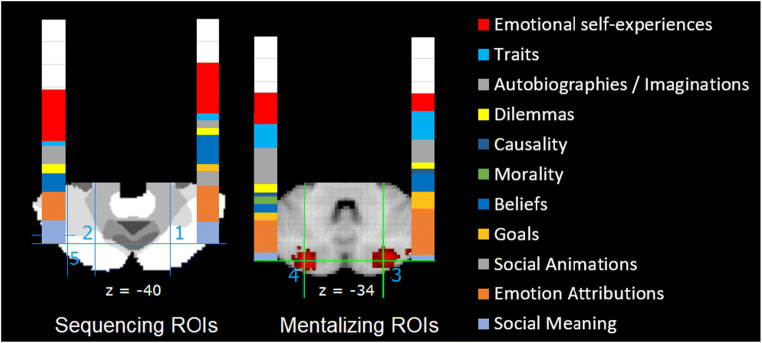
Results of the meta-analysis for distinct mentalizing task subcategories in proportion to 100% of all identified studies. All other nonmentalizing functions are denoted by white. ROIs 1 and 2 with MNI coordinates ± 25, − 75, − 40 are superimposed on the 7-network parcellation from Bruckner et al. [12], where the white area reflects the mentalizing/default network; ROIs 3 and 4 with MNI coordinates ± 26, − 84, − 34 are from the mentalizing meta-analysis in NeuroSynth

To investigate which areas of the posterior cerebellum are specialized for social mentalizing, Van Overwalle, Ma, and Heleven [119] isolated a number of regions of interest (ROI) which are frequently recruited during social mentalizing. Two “sequencing” ROIs were derived from recent fMRI studies that investigated a key aspect of cerebellar mentalizing: generating the correct sequence of social events that require the understanding of a person’s beliefs (Fig. 3, left panel). ROI 1 was isolated from a fMRI study that investigated the generation of social action sequences ([71]; *n* = 73) and was also identified in earlier research [24, 39]. ROI 2 was its left mirror location. These ROIs are quite close to two peaks reported in the meta-analysis by Guell et al. ([84]; about 6–8 mm away). Two additional ROIs (Fig. 3, right panel) were extracted from the automated “mentalizing” meta-analyses in NeuroSynth (i.e., from the 50 topics from the abstracts in the NeuroSynth database as of July 2018; see [13]). All four ROIs were located in lobule Crus II, in the mentalizing network demarcated by Buckner et al. [12]. All functional MRI studies in NeuroSynth within a radius of 6 mm around the coordinates of these four ROIs were selected, on the condition that they fulfilled a number of relevance/validity criteria such as coordinates expressed in MNI template, involving unmedicated healthy participants, the provision of an adequate control condition, and so on.

Each selected fMRI study was categorized in distinct mentalizing and nonmentalizing subcategories on the basis of the stimulus material of the main and control condition, using similar criteria as in earlier meta-analyses [2, 5, 7]. The results in Fig. 3 convincingly demonstrate that “social mentalizing” was the major functional category involving about 75% of the studies (when also including emotional self-experiences). In mentalizing tasks, the highest percentages were found in subcategories on attribution of other’s emotions (27%), emotional self-experiences (17%), autobiographies or imagined situations (16%), other’s beliefs (14%), other and self-traits (12%), and spontaneous social meaning in a human context (12%). To provide a comparative base rate of each task category, the NeuroSynth database was queried (extracted in July 2018; [13]) and revealed that mentalizing task categories ranged under 20% of all fMRI studies in NeuroSynth. This indicates that the high incidence of mentalizing studies in the selected ROIs in the mentalizing network of Buckner et al. [12] does not result from a higher base rate in general, but is specific to these areas.

Together, the meta-analysis by Van Overwalle, Ma, and Heleven [119] demonstrated that domain-specific social cognition related to social mentalizing and self-related emotional cognition is supported in selected areas of the cerebellar Crus II, with an incidence of about 75% on average. This points to a highly specialized area for social mentalizing processes in Crus II. These social mentalizing functions involve a broad range of explicit inferences about the mental state of other persons or the self, or inferences that are implicitly given in the social context of humans. The origin of the ROIs suggests that the slightly more anterior “sequencing” areas capture more the sequential nature of social cognition, while the more posterior “mentalizing” areas capture somewhat better social understanding that does not necessarily rest on a sequential order of action. In addition, recent dynamic causal modeling (DCM) analyses demonstrated that the “sequencing” ROIs are effectively connected by bidirectional loops to each of the bilateral TPJ, which are key parts of the mentalizing network in the cortex responsible for perspective switching [23, 24].

### The Role of the Cerebellum in Understanding Social Sequences: Evidence from Cerebellar Patients Studies and fMRI Research (Elien Heleven, Frank Van Overwalle)

A plethora of studies identified specific cortical regions involved in social understanding, such as the mentalizing network (for reviews, see [5, 120]). Unfortunately, the cerebellum was often neglected in these studies. Recently, however, a meta-analysis identified cerebellar involvement for several social tasks in healthy participants [2] and other studies documented strong connections between social processing regions in the cerebellum and cortex [23, 39, 70].

The cerebellum has traditionally been related to motor processes, where internal models are assumed to be responsible for the construction, detection, and application of motor sequences. To explain the involvement of the cerebellum in nonmotor processes, Leggio and Molinari [8] put forward the “sequence detection hypothesis” which states that the cerebellum evolved during human evolution to a similar function for purely mental sequences, based on frequently processed temporally or spatially structured sequences of events, including social events (see also contribution on “The sequencing hypothesis of the social cerebellum” by Leggio).

The role of sequencing in social understanding has typically been investigated using tasks in which elements of a sequence of actions need to be put in the correct chronological order. Leggio et al. [121] were the first to demonstrate decreased sequencing performance on verbal or pictorial action events among cerebellar patients compared to control participants, irrespective of the patients’ lesion type or location. A similar study reported reduced performance for cerebellar patients with isolated ischemic lesions as compared to healthy controls on sequencing tasks, especially when the sequences involved biological movements [122]. In yet another study, participants had to judge whether personal events (e.g., fetch parents at the airport) were ordered correctly or whether they really happened [123]. Among other regions in the cortex, the left posterior cerebellum was activated during order judgments, but not during reality judgments. All these studies demonstrate cerebellar involvement in social sequence processing. However, they investigated mainly basic social understanding through action observation in the present or past, not higher-level social processing.

**Fig. 4:**
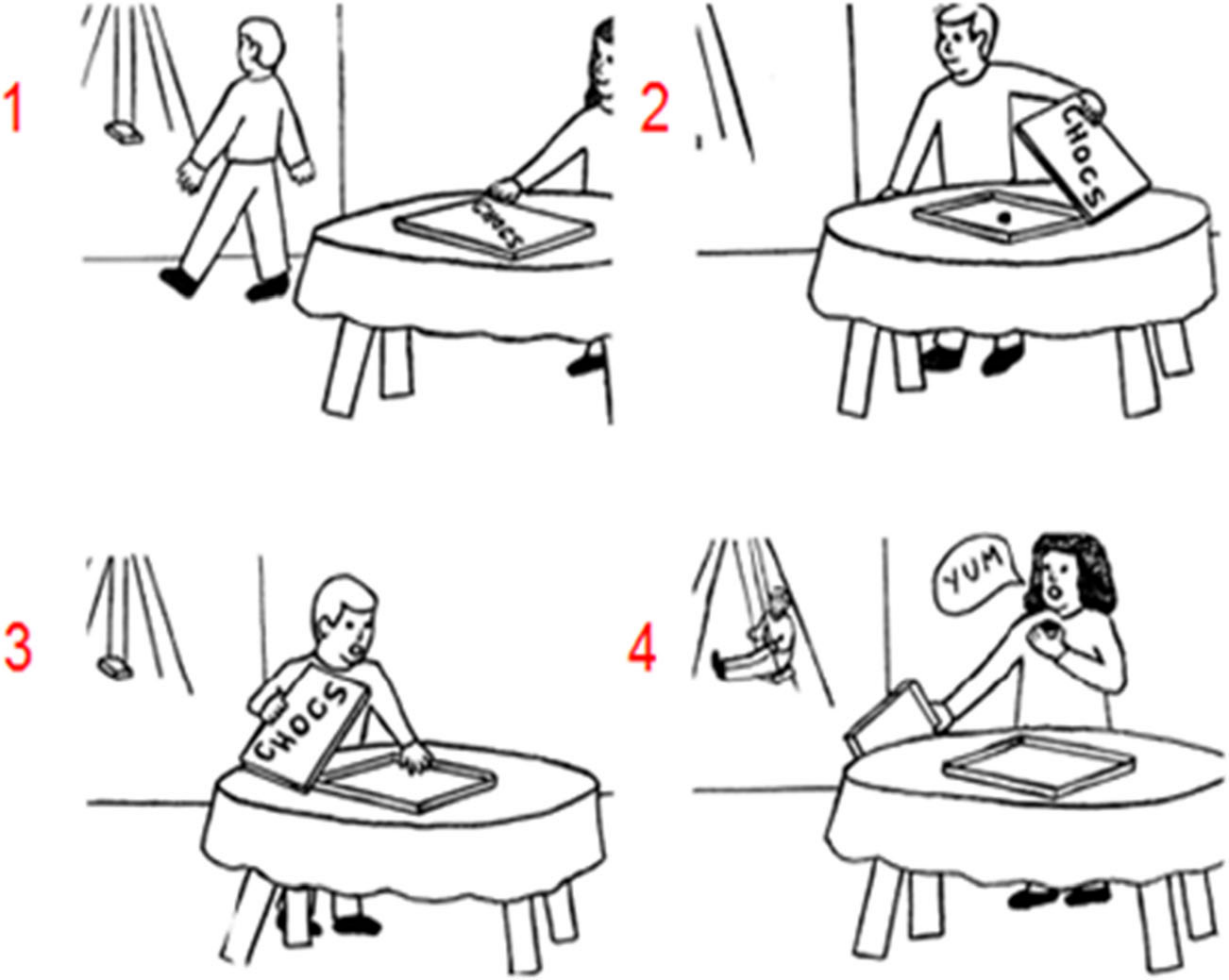
An example of a social false belief sequence in the picture sequencing task ([76]; the correct order is 2–1–4–3; the numbers are not shown to the participants but given here for display purposes). Participants had to select, in the correct order, the first picture on the screen, then the second picture, and so on

Higher-level social sequence processing requires the understanding of unobservable mental states of a person, and this process is termed “mentalizing” (for a review, see [4]). A recent pilot study investigated the role of mentalizing in the cerebellum [3] by comparing sequencing performance between healthy participants and patients with primary neurodegenerative ataxia or injury in the cerebellum on the picture sequencing task [76]. This task involves nonsocial routine mechanical events (e.g., a heavy wind knocks over a vase which falls on the ground), routine social scripts (i.e., an agent is shopping in a grocery store, goes to the cashier, and pays), and nonroutine social stories requiring the understanding of the mental state of an agent involving a false belief (see Fig. 4 for an example). In order to understand a false belief, participants need to infer a belief of an agent that is not conforming to reality (and hence is “false”). The patients ordered false belief events significantly less accurately compared to healthy control participants, but performed similar on other types of sequences. The authors concluded that the cerebellum is crucial for understanding social sequences involving false beliefs, and not (or less so) for routine sequences (i.e., mechanical and social scripts sequences) as they might require less inferences on the agents’ mental states.

**Fig. 5:**
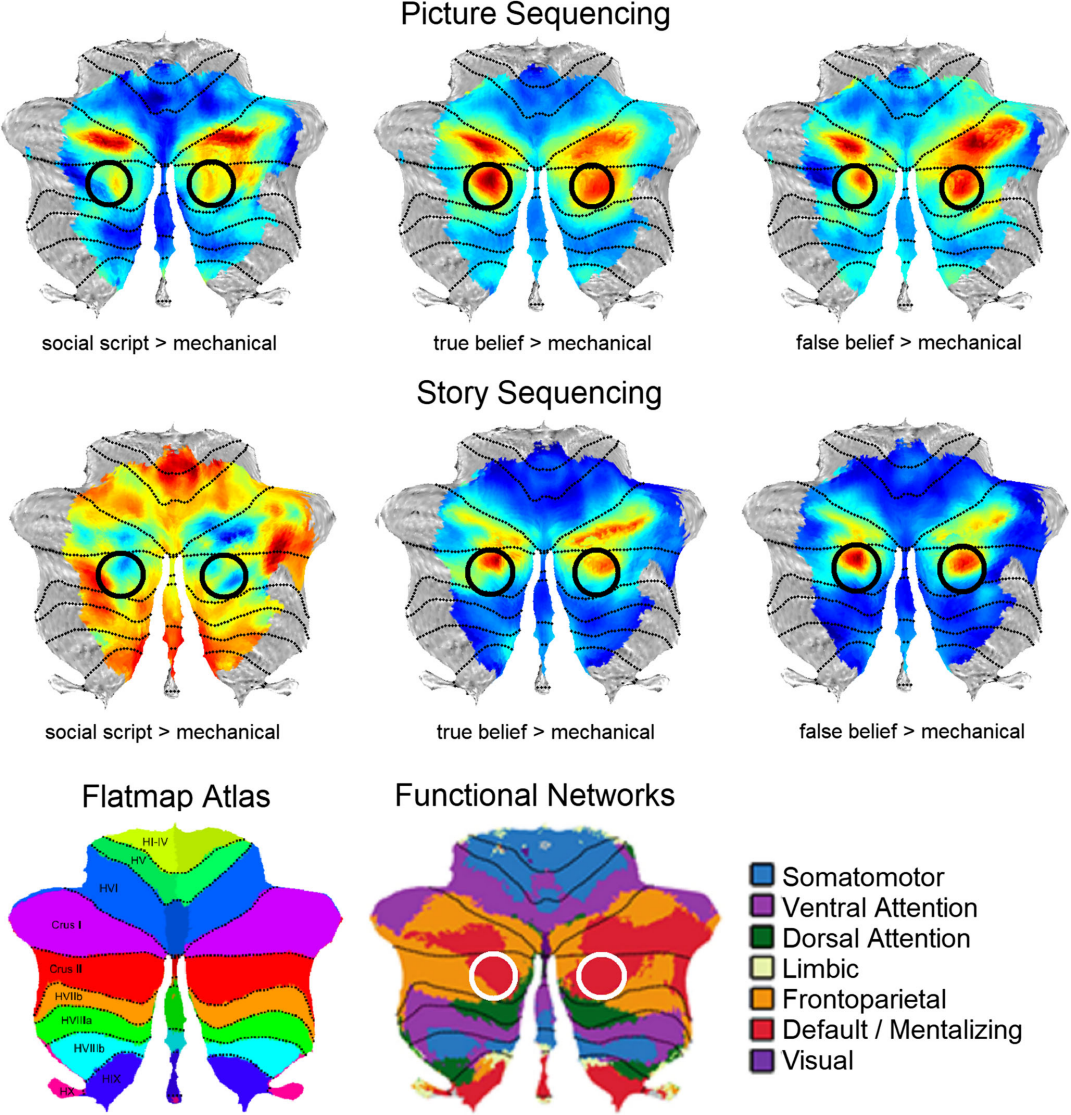
Top: Activation in the posterior cerebellum in the Picture and Story sequencing tasks for social scripts, true and false belief > mechanical comparisons shown on a SUIT flatmap [124] without threshold. True and false beliefs strongly activate Crus II in the default mode/mentalizing network, while social scripts activate this area in Crus II less so. Bottom: SUIT flatmap atlas showing the cerebellar lobules from [124] and functional networks from Fig. 2 of this consensus paper

However, this last study could not determine whether the novelty or false belief aspect of these stories was critical for cerebellar involvement. This issue was investigated in healthy participants by Heleven, van Dun, and Van Overwalle [71]. They extended the picture sequencing task with new social stories that included true beliefs. This enabled them to directly compare false versus true belief sequences. Moreover, they additionally developed a verbal version of the task. The results revealed significantly more activation in the posterior cerebellum (i.e., Crus I and II) during false and true belief event sequencing as compared to nonsocial mechanical event sequencing on the right hemisphere for pictorial sequences, and bilaterally for verbal stories (see Fig. 5). There was no difference in activation for false and true beliefs. These results demonstrate that novel beliefs in general, rather than false beliefs in particular, are a key aspect for cerebellar involvement. This might be due to the fact that the false and true stories in this study were closely matched and hence structurally very similar, so that they might have been approached in a similar manner. However, note that most belief studies compare false beliefs against nonsocial stories (see meta-analyses by [4, 5, 120, 125]), and only a couple of studies demonstrated larger activity for false than true beliefs [126–128]. A connectivity analysis, on the data of the picture sequencing task, revealed strong connections between the identified cerebellar areas and key mentalizing regions in the cortex [24].

We conclude that there is increasing evidence for cerebellar involvement in the understanding of social action sequences. However, research on this topic is still in its infancy. More research on healthy participants and cerebellar patients is needed. These studies should investigate whether specific regions within the (posterior) cerebellum can be exclusively linked to processing specific types of (social) sequences (e.g., new vs. routine; requiring belief inferences vs. not) or specific modes (e.g., verbal vs. pictorial). Given the low number of stimuli in some of the most promising tasks (e.g., picture sequencing; 4 stories per condition), developing more stimuli is also an important goal for future research.

### Explicit and Implicit Learning of Social Mentalizing Sequences (Min Pu, Qianying Ma, Frank Van Overwalle)

It has become evident that the posterior cerebellum plays a significant role in social mentalizing, including inferences on the intentions, beliefs, and traits of other people [2, 23, 70, 88]. But what is its function? Starting from the traditional view that the cerebellum plays a fundamental role in acquiring and predicting sequences in motor processing, Leggio et al. [121] and Van Overwalle, De Coninck, et al. [3] proposed that the cerebellum plays a critical role in reasoning on sequences of social actions. To test the role of the cerebellum on sequences in a social context, participants were given cartoon-like pictures or photos of human actions in a random order, and they were instructed to reconstruct the ordering of these pictures in a plausible sequence. The results showed that cerebellar patients were significantly impaired compared to healthy controls in correctly ordering human movements [121, 122] as well in ordering actions that require social mentalizing [3]. Critically, this last study found that deficits were largest during false belief stories which are a key determinant of mentalizing. The role of the posterior cerebellum in mentalizing about false and true beliefs during a picture sequencing task was confirmed in an fMRI study [71].

Recently, two novel sequencing tasks were developed to probe the breadth of the social function of the posterior cerebellum. These tasks involve social mentalizing in the context of explicit sequence learning [129] and implicit sequence learning [130].

#### Explicit Action Sequencing During Trait Attribution

**Fig. 6:**
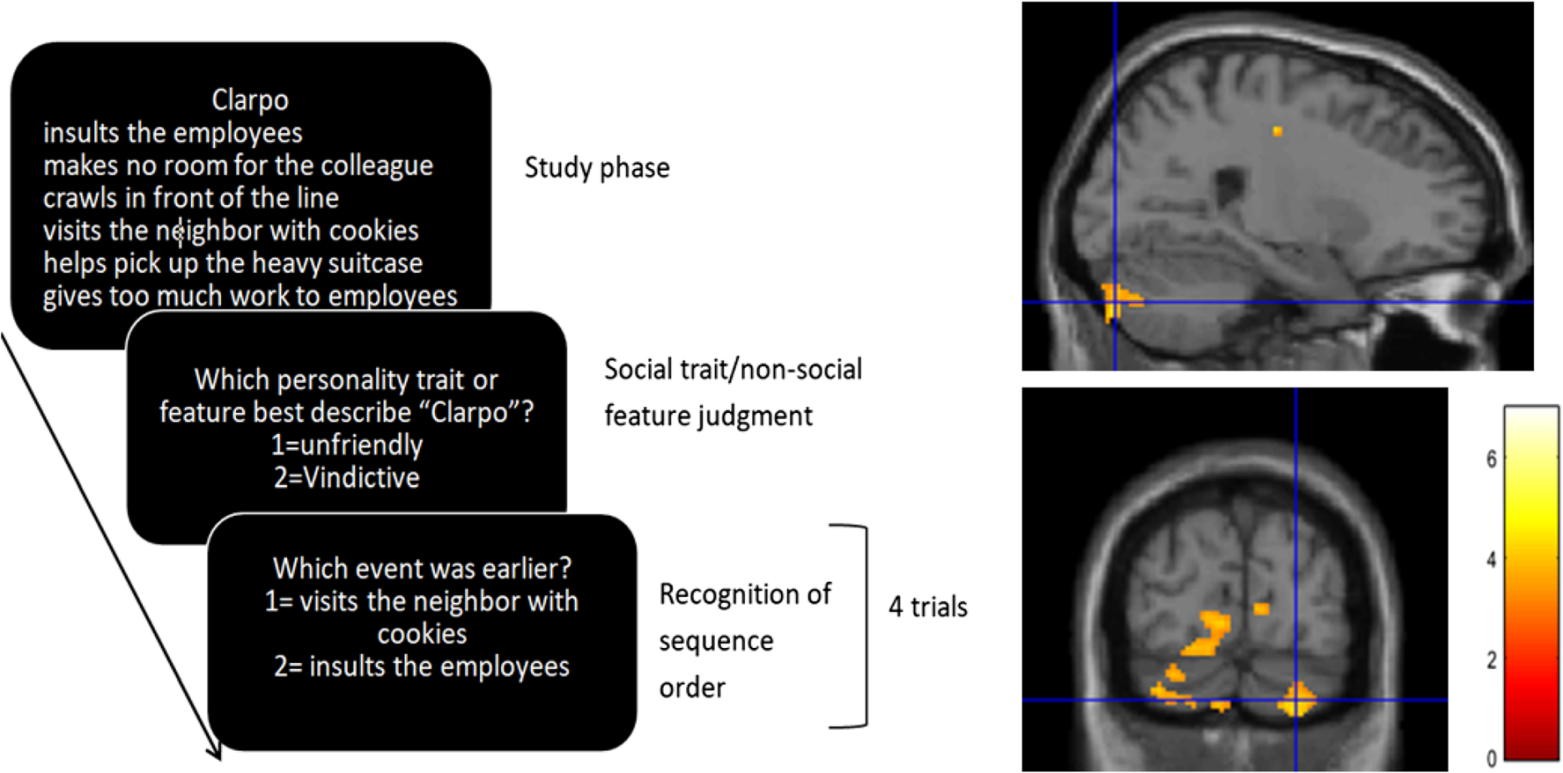
Left: Experimental procedure (abridged). Participants were instructed to learn the given temporal order of a set of six sentences involving a single person or object, and had to infer from these six sentences a common trait of the person or feature of the object. Right: A preliminary analysis comparing social (person) and nonsocial (object) conditions during this study phase revealed activation in the bilateral posterior cerebellum (MNI coordinates 20, – 76, − 36; *n* = 19)

Trait attribution reflects the question: what kind of person is this? This inference rests on the ability to integrate multiple behaviors in a single judgment about the person, and is crucial for social understanding, prediction, and interaction. The integration of action sequences to arrive at a single trait attribution may require a role of the cerebellum. However, prior research on the role of the cerebellum such as the picture sequencing task [3] was limited to sequences that implied a single goal or action. In contrast, trait attributions often integrate sequences of several actions on a larger time scale and across different social contexts. For instance, giving a compliment, buying a present, listening to someone, and so on are all distinct actions, but they are related by the same implied trait of kindness. To investigate the role of the cerebellum in learning action sequences during trait attribution, in a recent experiment, participants had to learn a given temporal order of various trait-implying actions and had to infer the trait implied by the behavior (see Fig. 6; [129]). Social sequence learning was interleaved with nonsocial sentence sets which implied a feature of an object. Preliminary fMRI data showed that the posterior cerebellum was more strongly activated when learning the order of trait-implying sentences in comparison with nonsocial sentences (see Fig. 6).

#### Implicit Sequences of True and False Beliefs

All previous tasks involved explicit instructions to provide the correct order of social actions. But what about implicit learning? People are able to process false beliefs at an implicit level, even at a younger age [131–133]. However, can people learn also sequences of true and false beliefs spontaneously and without realizing the occurrence of sequence learning? This question has seldom been asked in behavioral and neural approaches to social mentalizing. To study this process, a novel implicit mentalizing *serial reaction time* task was developed. In a classic serial reaction time, a target appears at one of four spatial locations and participants have to respond to each target’s location by pressing one of four keys [134]. However, unbeknownst to the participants, the target location follows a specific sequence of locations and participants appear to be able to learn the sequence without explicit knowledge of it.

**Fig. 7:**
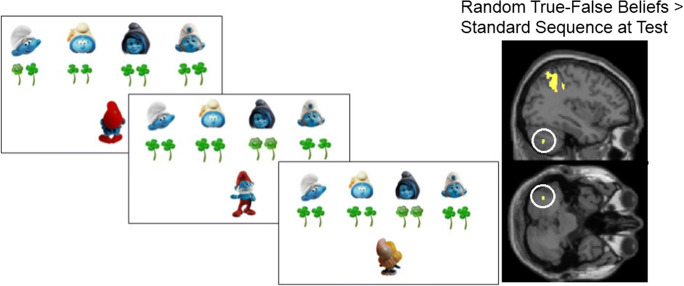
Left: The serial belief reaction time task. In this design, on each trial, participants had to report how many green flowers were received among green clovers according to one of two smurfs (i.e., Papa Smurf or Smurfette). On true trials, the smurf was turned to the screen and participants should report what the smurf could observe (the number of flowers); on false trials, the smurf was turned away from the screen and participants should report what they believed that the smurf saw last. Participants implicitly learned the fixed (but unknown) sequences embedded in the task, in particular the sequence of true and false beliefs. Implicit learning was attested by interspersing blocks with random instead of fixed standard sequences (e.g., random true–false beliefs), and observing significantly increasing response times as a consequence. Right: In a follow-up fMRI study [135], a parallel increasing pattern of posterior cerebellar activation during true–false belief randomization was observed (MNI coordinates − 36, − 64, − 42; *n* = 18)

In a belief version of the serial reaction time task [130], participants viewed one of two agents that repeatedly received flowers from four smurfs at four fixed locations on top of the screen (Fig. 7, left panel). Participants had to report how many flowers among likewise distractors were given according to the agent. Importantly, the agent was either oriented toward these locations and could see the flowers offered (i.e., true belief: the agent’s belief conformed to reality) or not (i.e., false or outdated belief: the agent does not know how many flowers are now given). In a false belief trial, the correct recollection of an agent’s last observation (when the flowers were last seen) led to the accurate response. The inclusion of two agents was essential to ensure that participants inferred a mental state associated with each agent independently, which is a necessary precondition for making a belief attribution [136]. A fixed sequence related to the two agents (Papa Smurf or Smurfette) and their belief orientations (true or false) was surreptitiously embedded and repeated in the task. Note that the motor response (i.e., how many flowers) was essentially random and independent from any of these implicit sequences. The results showed that participants implicitly learned the sequence of the agent’s true–false belief orientation in this social context, as revealed by increased response times when the learned true–false belief sequence was changed into a random belief sequence.

A follow-up fMRI study [135] using the same task revealed the role of the posterior cerebellum in this implicit belief sequence process. In parallel with the behavioral study, the results showed that activation in this area increased when the learned belief sequence was suddenly randomized (Fig. 7, right panel). This suggests that the posterior cerebellum also plays a role in implicit sequence learning of social beliefs, just like it does for explicit social sequence learning [3, 129].

## The Cerebellum and Body Reading

In this section, Marco Michelutti and Arseny Sokolov provide an overview of research on nonverbal body movements (e.g., by point-lights) and symbolic geometric shape animations. Chiara Ferrari and Zaira Cattaneo discuss the causal role of cerebellar regions involved in biological motion perception, and applied TMS at different time points to assess the timing of the cerebellar processes.

### Cerebellar Contributions to Nonverbal Social Cognition (Marco Michelutti, Arseny Sokolov)

From the time we recognize an approaching person by the way she moves, to when we ask ourselves if she would be bothered by our greetings, we constantly infer the intentions and goals from nonverbal cues such as body language. The processing of bodily expressions is thus a crucial prerequisite for mentalizing and adaptive social behavior [137–139]. Inferences on body language have been studied using both full-light and point-light body motion (BM). The latter consists of moving dots forming a schematic human figure and allows studying the effects of kinematics irrespective of other, potentially confounding information available in full-light displays [140]. Nonverbal social cognition has also been investigated through seemingly social interactions of abstract geometric shapes: Heider-and-Simmel animations require the observer to distinguish between animate and random motion patterns [141], while Frith–Happé animations introduce an additional level of mentalizing complexity (one shape seems to “read” the mind of the other) [142].

The neural correlates of the visual processing of point-light and full-light BM with and without straightforward intentions, as well as of social animations, largely converge within a widespread circuitry, with the right superior temporal sulcus (STS) and temporo-parietal junction (TPJ) as its key integrators [7, 143–146]. These shared neuroanatomical substrates further indicate that body language reading and mentalizing are intertwined. Indeed, mentalizing and social interpretations in the TPJ [147, 148] depend on multimodal input including information on the actions of others afforded primarily by the adjacent posterior STS [137, 146, 149]. High-resolution imaging analyses [150] including assessments of effective connectivity may yield additional insights on the functional organization, segregation, and crosstalk of the STS and TPJ underpinning body and mind reading.

The lateral posterior cerebellum has also been shown to contribute to nonverbal social cognition. Lesion data indicated that the left lateral cerebellum is indispensable for visual perception of point-light BM [151], and neuroimaging showed that the left lateral lobule Crus I implicated in processing of point-light BM entertains direct effective [17] and anatomical [16] connectivity with the right STS. A meta-analysis of 350 brain imaging studies suggested that the observation of full-light goal-directed BM does not only activate the posterior lobules Crus I and II, but also the anterior cerebellar lobuli, primarily involved in sensorimotor processing [2].

For abstract social animations, no consensus has been reached yet as to the precise cerebellar structures involved. While the left posterior lobule VIII has been put forward in a meta-analysis [2], other studies have rather pointed to activation of the bilateral lobules Crus I, Crus II, and VIIB [18, 84, 146], a topography more similar to that involved in body language reading. Specific effective connectivity between the left lobule Crus II and the right STS was also seen during passive viewing of these animations [18]. Furthermore, activation of a cluster in the left Crus I has been related to greater propensity to attribute intentions to the shapes [18].

In patients with early behavioral variant frontotemporal dementia, reduced (but not atrophic) gray matter in the right Crus I and II and the right vermal lobules IX and X was associated to impaired attribution of intentionality in Frith–Happé animations [152]. Deficient processing of abstract animations [153] and of BM [154, 155] have also been observed in schizophrenia and autism spectrum disorder (ASD). The degree of social impairment in ASD was related to the regional activation of the left lobules VI and Crus I as well as of the right Crus II during visual processing of BM [15]. In the same study, aberrant effective connectivity of the right posterior STS with the bilateral lobules VI and the right Crus I/II during visual perception of BM was also associated with altered social behavior. Furthermore, reduced activity in the left Crus I during the attribution of mental states to Frith–Happé animations was reported in individuals with ASD [156].

Overall, the imaging and clinical data point to involvement of the lobules Crus I and II and their interaction with the STS in social inference based on nonverbal dynamic information. The lateralization to the left cerebellum and right STS appears more evident for BM processing than for abstract social animations. Recent theoretical reasoning and functional MRI data indicated that functional zones in the cerebellum may be better conceived as stripes or clusters crossing the conventional lobular boundaries [21, 124]. A functional rather than anatomical parcellation may thus better represent cerebellar subdivisions. In this respect, meta-analytic connectivity [88] suggested that the cerebellar clusters involved in visual processing of BM and social animations overlap with the mentalizing network of the 7-network resting-state functional connectivity parcellation of the cerebellum [12].

The cerebellar contributions have been shown to be specific for socially significant motion, rather than dynamic stimuli in general. Specificity of the left lateral cerebellar involvement in processing BM was demonstrated by comparing canonical to scrambled [17] and inverted point-light BM [151]. Activation in the left Crus II was found to be higher for goal-directed than nonintentional gestures [157]. The cerebellar clusters involved in processing of abstract social animations appear to be more sensitive to dynamic shapes exhibiting complex mental states than to those moving randomly [152, 156, 158]. Interestingly, the levels of activation in bilateral Crus I [158] and right Crus II [152] for shapes that represent explicit goal-directed agency (e.g., following) were shown to be intermediate, i.e., lower than for complex mental states, but higher than for random motion.

While these findings suggest that the cerebellum plays an important and specific role in the interpretation of nonverbal cues for social perception and communication, determining the nature of its functional contribution to the underwriting brain circuitry requires additional research. The cerebellum is increasingly considered to contribute to the generation, online-matching, and adaptive fine-tuning of internal models [21]. Adaptation of internal models relies on prediction errors, i.e., the difference between predicted and actual outcomes, and may be crucial for both sensorimotor processing and cognition [21, 159]. Prediction errors during a semantic processing task were found to activate bilateral Crus I and II [160]. Prediction errors are also a central element in the active inference framework. This is a recent computational and neurobiological account for the adaptation of internal models for action, perception, and cognition [161]. Active inference refers to the Bayesian (probabilistic) minimization of prediction errors through a reciprocal, hierarchical exchange of information between neural populations. Bayesian simulations also afford plausible approximations of human inferences on the mental states of others [162], suggesting that active inference may underwrite social cognition [62]. Surprisingly, inclusion of subcortical structures, such as the cerebellum, into the active inference framework has begun only recently [163], although it may substantially benefit conceptual reasoning and empirical research. The lateral posterior cerebellum might support the STS, TPJ, and other structures in the cerebral cortex in the comparison and adjustment of social models to actual internal and external outcomes through detection and signaling of violated predictions, as well as event sequencing [20].

In a nutshell, we suggest that the adaptive prediction within sociocognitive cerebro-cerebellar loops may facilitate active social inference. Given the rather uniform structure of the cerebellum, the cerebellum might afford similar computations across the different subdomains of social cognition. Data on impaired interpretation of verbal faux pas stories in patients with left posterior cerebellar degeneration may point toward adaptive active inference and prediction as a common denominator of cerebellar contribution to verbal and nonverbal social cognition [38]. However, this hypothesis remains to be confirmed by specifically designed experiments and computational models across verbal and nonverbal social cognition. A multimodal brain imaging and interdisciplinary, translational, and clinical assessment of the various sociocognitive cerebro-cerebellar loops is required to elucidate whether they are recruited in a domain-specific or a domain-general fashion [164, 165].

### TMS and Cerebellar Regions Involved in Biological Motion Perception (Chiara Ferrari, Zaira Cattaneo)

One of the mechanisms by which the cerebellum may contribute to social cognition is the processing of biological motion. Indeed, the human ability to perceive biological motion, that is movement patterns made by other individuals (but also by animals), is critical for successful social behavior and nonverbal communication (e.g., [137]).

Using point-light displays [140] in an fMRI study specifically focusing on the cerebellum, Sokolov et al. [17] observed increased activity in response to point-light upright walker animations compared to scrambled-walker animations selectively in the left posterior cerebellar hemisphere, specifically in Crus I and lobule VIIB [140]. The preferential activation in the left cerebellar hemisphere is likely to reflect the predominant role of the right (vs. left) superior temporal sulcus (STS) in processing biological motion, with which the left posterior cerebellum (left lobule VI, left Crus I/II) has been found to be anatomically and functionally connected (e.g., [14–18, 143]). Moreover, in considering these findings, it is important to stress that point-light walker stimuli are typically represented as walking on the spot (this was indeed the case in [140]). Accordingly, these stimuli might not fully represent a goal-directed action (in fact, the main goal of the locomotor function is to displace the whole body from one position to the other) and may thus not be able to induce the same motor resonance/mirror mechanism expected when observing natural locomotion (e.g., [166]). This may account for the lack of significant anterior cerebellar activations during biological motion processing in point-light displays. Indeed, as mentioned in the introduction, the anterior cerebellum (as part of the sensorimotor network) is likely to be preferentially recruited by motion-related mirroring tasks (see [2]; for some recent empirical evidence, see [167]).

Whereas neuroimaging evidence is only correlational, patients’ studies provide a causative approach to the study of function–structure relationships. Interestingly, Sokolov et al. [151] showed that patients with (left) posterolateral but not anteromedial cerebellar lesions showed impaired visual sensitivity to the presence of a walker in point-light displays, supporting neuroimaging evidence in pointing to a role of the posterior cerebellum in mediating biological motion perception. However, the lesion approach is hampered by the issue of compensatory plasticity and by the possibility that the disturbance to function may be more or less widespread than the anatomical lesion. These limits may be overcome by mimicking a real lesions’ effect using noninvasive brain stimulation (the “virtual lesion” approach, see contribution “Targeting the social cerebellum by noninvasive neurostimulation” by van Dun and Manto). In particular, transcranial magnetic stimulation (TMS) can be employed to selectively target different cerebellar regions.

In a recent study employing TMS [168], we assessed whether the left posterior cerebellar lobe *causally* contributes to biological motion perception. Healthy adult volunteers were presented with point-light animations depicting a biological figure in motion (performing various activities such as walking, kicking, or throwing) that they had to discriminate from nonbiological motion animations (scrambled versions of the original stimuli, in which dot positions were randomly modified while preserving their kinematics). Triple-pulse 20 Hz TMS was delivered concurrently with the task (i.e., online) over both medial (vermal lobule VI) and lateral (left lobule VI/Crus I) sectors of the posterior cerebellar lobe, or over the vertex as a control site. We found that TMS delivered at the onset of the animations over the posterior vermis, but not over the left posterior cerebellar hemisphere, interfered with participants’ ability to distinguish biological from scrambled motion compared to vertex (control) stimulation. Interestingly, when stimulation was delivered at a later time point (300 ms after stimulus onset, following STS response to biological motion, e.g., [169]), participants performed worse when TMS was delivered over the left cerebellar hemisphere compared to the vermis and the vertex. These data provide some preliminary evidence for a *causal* role of medial and (left) lateral sectors of the posterior cerebellum in mediating biological motion processing. One hypothesis of the functional significance of the cerebellar contribution to sensory processing is that the cerebellum controls the acquisition of sensory data, thus indirectly facilitating the computational efficiency of the rest of the brain ([170]; see [171] for a commentary). This explanation is consistent with the disrupting effects of vermal TMS on other visual discrimination tasks, such as discrimination of (nonbiological) motion direction [172]. An alternative hypothesis is that the cerebellum aids visual information processing by making predictions in the form of internal models of sensory events ([173]; see [171] for discussion). Interfering by means of TMS with activity in lobule VI might have affected the monitoring of incoming visual information or the implementation of internal models (prediction of sensory outcomes) important for recognition of biological motion. Moreover, our findings underlined functional differences depending on the anterior/medial-to-posterior/lateral gradient in lobule VI, in that the more anterior medial lobule VI appears to modulate early movement identification, while the more posterior lateral Crus I is likely to modulate later higher-level cortical processing. Accordingly, we have recently demonstrated [174, 175] that TMS over the left posterior cerebellar hemisphere also affects discrimination of emotional facial and body expressions (shown in static pictures of real-life individuals), converging in pointing to the *causal* role of this posterolateral region in inferring others’ mental states from observation of their body language.

Further TMS evidence is needed to clarify the possible *causal* contribution of different cerebellar regions (both in the anterior and posterior cerebellum) to biological motion processing and, more broadly, to action understanding tasks that require increasingly less mirroring and increasingly more mentalizing processes.

## The Cerebellum and Clinical Aspects

In this section, Silvia Clausi, Michela Lupo, and Maria Leggio provide an overview of the clinical implications of the cerebellar role in mentalizing, which could underlie the difficulties in social cognition reported in cerebellar patients as well as in individuals with autism. Giusy Olivito, Libera Siciliano, Frank Van Overwalle, and Maria Leggio report on the connectivity within cerebello-cerebral mentalizing networks and their clinical implications. Next, Laura Rice and Catherine Stoodley focus on the role of the cerebellum among individuals with autism. They discuss research with animals and humans on cerebellar structural and functional connectivity to elicit the origin and consequences of cerebellar abnormalities in autism.

### Clinical Implications of the Cerebellar Role in Mentalizing (Silvia Clausi, Michela Lupo, Maria Leggio)

The mentalizing process is part of the social cognition ability and refers to the capacity to understand other people’s mental states (like their beliefs, intentions, emotion) and adopting the perspective of others to understand and predict their behavior. Several studies described impairments in social functioning and mentalizing process in psychiatric (i.e., mood disorders or schizophrenia) and neurodevelopmental (i.e. autism) disorders and in neurological illnesses (i.e., Alzheimer’s, Parkinson’s, or cerebellar disorders), that affect their daily life in a very profound way.

The involvement of cerebello-cortical networks in the mentalizing process opens up new perspectives in clinical practice when treating patients with neurodegenerative, psychiatric, and neurodevelopmental disorders. In a recent study, Clausi and colleagues [38] demonstrated that patients affected by degenerative cerebellar pathology were impaired in different social cognition abilities. Their impairment was linked to gray matter reduction localized in specific portions of the cerebellum (vermis and bilateral Crus I/II). Intriguingly, these areas showed decreased functional connectivity with cerebral areas involved in mirroring and mentalizing processing [38, 176].

These findings support previous suggestions that the altered cerebellar functional modulation of cerebral projection areas involved in emotional and mentalizing processing [2, 108] could underlie the social cognition difficulties reported in patients who present structural or functional alterations in the cerebellum. Indeed, a defective mentalizing process has been described not only in patients with cerebellar ataxia [38, 108] but also in patients with different neurodegenerative disorders [177] in which cerebellar alterations are described, such as Alzheimer’s and Parkinson’s diseases [178, 179]. Of relevance, an association between mentalizing impairments and specific patterns of abnormal connectivity between the left inferior temporal gyrus and lateral prefrontal and cerebellar areas was reported in myotonic dystrophy type 1, a genetic multisystemic disorder in which the brain is one of the several involved organs [180].

In the clinical field, mentalizing ability was largely described as being impaired in individuals affected by psychiatric disorders such as schizophrenia and mood disorders [181–183] or by neurodevelopmental disorders such as autism spectrum disorders [184]. In line with this, in a recent study by our group, the mentalizing abilities of patients with degenerative cerebellar disease and adults with autism were directly compared. The results showed that both cerebellar and autistic participants failed to process the immediate perceptual component of the mental state recognition (i.e., to recognize the mental state of other people from the eyes expression) and the more complex conceptual level of mentalization (i.e., to recognize a false belief) [20, 185].

A similar pattern of alterations that may reflect impairment in the automatic or implicit processing of mentalizing was also described in patients affected by schizophrenia who failed to select the appropriate behavioral representation for understanding the actions and intentions of others [186, 187]. Moreover, mentalizing deficits seem to be a substantial feature of mood disorders, both in manic and depressive states [182, 183] and in remitted patients with bipolar disorder [188]. In a population of patients affected by type 1 or type 2 bipolar disorder who were in a euthymic state, preliminary data showed that patients exhibited specific mentalizing problems when they were required to understand another person’s mental state and consider their beliefs and intentions, although they maintain the ability to feel the emotional impact of a given situation (personal observations—manuscript in preparation).

Interestingly, together with this clinical evidence of social impairment, increasingly more neuroimaging studies have shown structural and functional alterations in the regions of the “social cerebellum” and in the cerebello-cerebral mentalizing networks in neuropsychiatric populations [77, 79, 189–194]. Indeed, in schizophrenia, evidence demonstrated microstructural disruption of cerebro-cerebellar pathways [195] and intracerebellar white matter [192]. Moreover, specific cerebellar alterations have also been described in other psychiatric conditions, such as mood disorders (see [196] for a review). In patients with bipolar disorder, reduced volumes and decreased activity in the vermis and posterior cerebellar lobes [191, 193, 194, 197–201] and reduced functional connectivity between the posterior cerebellum and amygdala, inferior frontal gyrus (orbital), and striatum were reported [198, 202–204]. Additionally, in adults with autism, lower resting-state functional connectivity between the left Crus II and the right temporo-parietal junction adjacent to the STS [205] and altered functional connectivity between the left dentate nucleus and the right cortical regions involved in social cognition [77, 79] were revealed.

To better highlight the cerebellar role in the socioemotional processing of specific psychiatric conditions, Lupo and colleagues [206] demonstrated a probable association between the onset of a manic state and the occurrence of an isolated cerebellar lesion in a single case study, finding a clear overlap between the patient’s impaired functional connectivity and the cerebello-cerebral mentalizing networks usually altered in bipolar disorder patients.

Taking into account the cerebellar and cerebello-cerebral circuit alterations found in these pathological conditions and the similar impairments in mentalizing process, it is evident that the study of cerebellar functioning in these pathologies could be useful at two levels. First, these data support the hypothesis that difficulties in social interactions and personal relationships described in neurodegenerative pathologies are a direct consequence of brain abnormalities and not a patient’s reaction to illness. This finding is relevant not only for a better comprehension of the neurobiological bases of social behavior impairment in neurodegenerative and psychiatric disorders but also for the implementation of nonpharmacological interventions (e.g., psycho-educational treatment, counseling or cognitive rehabilitation) to improve clinical aspects and impact on patients’ quality of life.

Second, the specific characterization of the cerebellar modulatory function on the cortical projection areas involved in mentalizing processes may help to develop specific rehabilitation protocols to modulate cerebellar excitability, allowing clinicians to influence/improve symptomatology in individuals suffering from mentalizing alterations. Among these innovative treatments, transcranial direct current stimulation (tDCS) should be mentioned.

### Connectivity Within the Cerebello-Cerebral Mentalizing Network and Clinical Implications (Giusy Olivito, Libera Siciliano, Frank Van Overwalle, Maria Leggio)

#### Cerebello-Cerebral Functional Connectivity Patterns in Relation to Mentalizing Functions

Over the last decade, cerebellar functional topography for emotional [207] and social processing [2, 88] has been well established. Indeed, beyond the classic distinctions between the sensorimotor and the cognitive cerebellum [118], it is now clear that the cerebellum plays a crucial role in emotional and social processing through functional interactions with cerebral regions. In the same way as motor and cognitive processing, emotional and social processing are topographically arranged within the cerebellum [208]. The understanding of anatomical connectivity with the cerebral cortex has provided substantial support for defining the functions of each of the cerebellar subregions [209], and in more recent years, advanced techniques assessing functional connectivity have proven very useful for cerebellar research aiming to establish a better understanding of segregated cerebellar functional organization, including emotional and social–cognitive domains.

Functional connectivity reflects the synchronous activation of spatially separated brain regions [210] and can be measured by detecting spontaneous fluctuations in brain activity during resting-state fMRI [211, 212]. Functional connectivity findings have consistently indicated that cerebellar zones have a domain-specific functional topography rather than domain-general executive and semantic support [2], playing specific roles through functional interactions with the cerebral cortex. According to functional connectivity studies on healthy participants [12], functional overlap has been suggested between cerebellar areas involved in social mirroring (i.e., “body” reading) and somatomotor networks, as well as between the mentalizing network of the cerebrum and the mentalizing network of the cerebellum [70]. In the context of mentalizing functions, the default mode network is of particular interest [213] since it includes a set of cerebral regions (i.e., the TPJ) that are particularly relevant for the social understanding of others [214, 215]. The cerebellar contribution to the default mode network has been evidenced in distinct resting-state fMRI studies [12, 216–219], showing that cerebellar Crus I/II are functionally coupled to default mode regions, while anterior Crus I is functionally associated with the cerebral frontoparietal network [220]. Thus, according to this functional segregation, Crus II is preferentially recruited when high-level social processing is in demand [39], and bidirectional (closed-loop) connectivity between this region and the bilateral TPJ is specifically related to high-level social understanding [23].

#### Cerebello-Cerebral Mentalizing Networks in Relation to Pathological Conditions

Since the first description of severe social and emotional impairment in patients with cerebellar damage was outlined [221], social neuroscientific research using fMRI has provided valuable insights into understanding the role of the cerebellum in social cognition. According to the abovementioned evidence, the study of the connectivity within cerebello-cerebral mentalizing networks has gained increased attention in the context of pathological conditions that differently affect the cerebellum. In line with the idea that cerebellar modulatory function underlies social cognition processes at different levels, decreased functional connectivity in multiple segregated cerebello-cerebral networks has been found in patients with cerebellar neurodegenerative pathologies who demonstrated impairment in both lower-level and complex conceptual levels of mentalization [38]. Hypoconnectivity between the cerebellum and specific frontal regions has been described in a 43-year-old female who presented significant impairment in social interaction following left-side cerebellar lesion [206]. Increasing evidence on the role of the cerebellum in the optimization of cognitive and affective functions, supported by cerebellar connections with supramodal association cortices and the limbic system, has led to the hypothesis of its involvement in the onset of social alterations in neurodevelopmental and psychiatric disorders [222, 223].

Additionally, fMRI studies have suggested that the dysregulation of cerebellar outputs to default network brain regions may be responsible for social impairment typically observed in individuals with autism spectrum disorder [77] and attention-deficit/hyperactivity disorder [224, 225].

Emerging literature in social cognitive neuroscience suggests the occurrence of mentalizing dysfunction in several psychiatric populations [226, 227]. In the context of psychiatric disorders, extensive evidence has pointed to mentalizing alterations in patients affected by schizophrenia [228–230], in which cerebellar impairments have also been reported [222, 231, 232]. Recently, Bora and Pantelis [227] suggested that mentalizing alterations may also affect social functioning in patients with bipolar disorder. A recent systematic review [196] identified several studies demonstrating disrupted functional connectivity between the posterior cerebellum and mentalizing regions (i.e., the TPJ, medial prefrontal cortex, posterior cingulate) in individuals experiencing a depressive state and in patients with a diagnosis of bipolar disorder [204, 233]. Likewise, decreased functional connectivity has been shown between the cerebellum and temporal and parietal regions in major depressive disorder [234].

The paucity of the empirical data collected thus far does not allow for a conclusive theory of the specific role of cerebellar–cerebral networks in mentalizing deficits in psychiatric and neurodevelopmental disorders. Further studies directly investigating cerebellar–cerebral networks and mentalization are needed to overcome this gap and shed more light on the role of the cerebellum in the mentalizing domain. Furthermore, as evidenced in the contribution on “Clinical implications” by Clausi et al., a better comprehension of the neural substrate of social behavior impairment in psychiatric and neurodevelopmental disorders may have important clinical implications. Perhaps, it may facilitate developing rehabilitation protocols specifically targeting the cerebellum (i.e., by transcranial direct stimulation) to improve clinical outcomes related to mentalizing processing in patients. Potential applications of cerebellar neurostimulation are discussed in the next sections of this consensus paper.

### Cerebellar Contributions to Social Behaviors and Social Networks in Autism (Laura C. Rice, Catherine J. Stoodley)

Autism is a neurodevelopmental condition characterized by atypical social interaction and communication together with repetitive behaviors and restricted interests [235]. Although an extensive network of regions underpins these complex behaviors, the cerebellum is consistently implicated in autism and has recently gained attention as a potential biomarker [236] and therapeutic target [237]. While the highest risk factor for autism is having an identical twin with autism, the second greatest risk factor for an autism diagnosis is early developmental damage to the cerebellum (see [238]), and early cerebellar damage is associated with increased internalizing behaviors, affective and attentional deficits, and withdrawal from social contact [239]. These data suggest that atypical cerebellar development could contribute to the characteristic behaviors associated with autism [240].

There are multiple lines of evidence that the cerebellum is involved in social cognition in neurotypical populations (see this consensus paper; [2, 165]). The cerebellum is part of frontoparietal (executive control), default mode (mentalizing), and limbic (emotion) networks (see [12]), which are critical to social communication and interaction. It has been proposed that the cerebellum contributes to social cognition by building internal action models to anticipate others’ actions and one’s response to such actions [20, 165], automatizing social interactions and rapidly detecting disruptions in action sequences (e.g., [71]). Developmental disruption of this system in autism could lead to a failure to acquire or automatize the information critical to efficient, effective social cognition.

Animal [241–245] and human [237, 246–252] studies have explored the role of the cerebellum in autism from cells to systems (for more comprehensive reviews, see [190, 238, 253, 254]). In a clustering analysis of 26 different mouse models of autism, all groups and models showed cerebellar abnormalities [255]. Atypical Purkinje cell structure and function have been reported in the tuberous sclerosis mouse model of autism (TSC1; [244]), and chemogenetic stimulation of Purkinje cells rescued social impairments in TSC1 mice [237], critically linking cerebellar dysfunction to core autism behaviors. As in humans, early developmental cerebellar disruption in rodents leads to autism-like behaviors [245, 256].

In humans, cerebellar abnormalities have been identified from the earliest neuroimaging studies of autism (e.g., [257]; for review, see [190]). Both gross differences in cerebellar volume (e.g., decreased cerebellar cortical volume; [250]) and reduced gray matter volumes in specific cerebellar regions have been reported (e.g., the posterior vermis and Crus I/II; [252]). A meta-analysis showed converging support for reduced gray matter in the inferior cerebellar vermis (lobule IX), left lobule VIIIB, and right Crus I in autism when compared with neurotypical cohorts [223]. Volumetric differences in the posterior vermis and bilateral Crus II [251] and right VI and Crus I/II [252] significantly correlated with social interaction and communication scores in children with autism, suggesting that these specific cerebellar regions may be involved in core autism behaviors.

#### Cerebellar Structural and Functional Connectivity in Autism

Further support for the role of the cerebellum in autism comes from structural and functional connectivity studies (for reviews, see [253, 258–260]). Early studies reported decreased cerebellar white matter density [261, 262] and larger cerebellar white matter volume in autism [263]. Diffusion imaging studies have revealed decreased fractional anisotropy (FA) and increased mean diffusivity (MD) in the middle and superior cerebellar peduncles, suggesting altered integrity of the pathways connecting the cerebellum with the cerebral cortex [264–267]. Several studies have reported reversed [268] or reduced [269, 270] lateralization patterns of FA in the cerebellar peduncles, mirroring the rightward cerebral cortical lateralization that has been reported in autism [271, 272]. These white matter differences are also behaviorally relevant: cerebellar white matter volume was a significant predictor of future autism diagnosis [273], and decreased cerebellar FA correlated with autism symptom severity [274].

Resting-state functional connectivity findings further suggest atypical cerebro-cerebellar networks in autism, consistently reporting reduced connectivity within established networks, especially in those relevant for social interaction. Reduced functional connectivity between right Crus I/II and regions of the “social brain,” including the medial prefrontal cortex and superior temporal sulcus, has been reported in autism (for summary, see [252]). A recent study revealed significant hypoconnectivity in autism between Crus I/II and lobule IX and areas supporting language (bilateral superior temporal gyrus and inferior frontal gyrus), emotional (amygdala), and social (default mode network) functions, and more atypical connectivity was associated with more severe scores on the Autism Diagnostic Observation Schedule [275]. Increased connectivity between nonmotor regions of the cerebellum and sensorimotor cerebral cortical regions has also been reported in autism, indicating atypical crosstalk between sensorimotor and nonmotor cerebro-cerebellar circuits ([276]; though see [277]).

#### Cerebellar Functional Activation in Autism During Social Task Paradigms

Atypical cerebellar activation has been reported in autism during a range of social tasks, from action observation to mentalizing (for reviews, see [165, 190, 238]). As with structural and connectivity data, differences in autism often involve lobule VII (including Crus I and II). For example, during the Frith–Happé triangle animations task, the autism group had more difficulty with the task and showed decreased activation in left Crus I [156], along with reduced functional connectivity between Crus I bilaterally and medial regions of the default/mentalizing network [2]. During a social judgment task, the neurotypical group engaged bilateral lobule VII (Crus II), while the autism group showed significantly less cerebellar activation (bilateral VI and VII) during the social condition relative to gender judgment [278]. In a study where performance did not differ between the autism and neurotypical groups, the autism group showed increased engagement of Crus I bilaterally during a causal attribution task, which may reflect a compensatory mechanism [279]. These findings suggest that successful engagement of the cerebellum may be critical to performance on a range of social measures.

#### Conclusion

In summary, there is converging evidence for atypical cerebellar (specifically lobule VII) structure, function, and connectivity in autism. These findings suggest that the cerebellum could be a potential target for therapeutic intervention to improve social outcomes in individuals with autism.

## Cerebellar Neurostimulation

This section focuses on possible ways to ameliorate social dysfunctions by cerebellar neurostimulation. Kim van Dun and Mario Manto discuss the social cerebellum as promising target of noninvasive neurostimulation in various impairments of social cognition, while Elien Heleven and Frank Van Overwalle provide preliminary evidence from a pilot study on the effect of cerebellar TMS on performance in social sequencing.

### Targeting the Social Cerebellum by Noninvasive Neurostimulation (Kim van Dun, Mario Manto)

Noninvasive brain stimulation such as transcranial direct current stimulation (tDCS) and transcranial magnetic stimulation (TMS) have been shown to modulate neural cerebral [280] and cerebellar excitability [281]. Both tDCS and TMS can be applied by using a wide variety of parameters. Studies applying tDCS over the cerebellum usually stimulate for 15–20 min at 2 mA bilaterally or unilaterally [282]. It is commonly accepted that the areas underneath the anode will be excited, while areas underneath the cathode will be inhibited, although the excitatory or inhibitory effect also depends on other factors such as neuronal orientation within cerebellar lobules and the multiple folia [283]. A recent meta-analysis indeed showed no clear polarity-specific effect of cerebellar tDCS [284]. We cannot exclude distinct responses between motor and cognitive/affective tasks on the basis of the topography of the circuits involved. In addition, the montage/current densities selected might also impact the effects observed with tDCS. Transcranial alternating current stimulation (tACS) instead of direct current may also be used, but research studies on cerebellar tACS are still scarce [285]. TMS is applied as a single pulse to study the neurophysiological effects of transiently disrupting/altering neural excitability in a specific area and can also be used repetitively (rTMS) to induce a longer-lasting effect. Typically, it is assumed that rTMS at low frequencies (~ 1 Hz) is inhibitory, whereas the effect at higher frequencies (≥ 5 Hz) is excitatory [286]. A more advanced TMS protocol is theta burst stimulation (TBS), which uses a burst of 3 pulses at 50 Hz instead of single pulses. These can be delivered in a continuous manner (cTBS), inducing inhibitory effects, or with intermittent pauses (iTBS), assumed to be excitatory [286].

Because of the location of the cerebellum in the posterior cranial fossa, the posterior part can easily be reached by tDCS or tACS, although higher intensities might be needed to penetrate the skull and reach sufficient electrical current density within the cerebellar cortex [287, 288]. For TMS, studies have shown that a double cone coil, together with higher stimulus intensities, might be needed to stimulate the cerebellum in a localized manner [289, 290]. However, it should be noted that cerebellar magnetic stimulation can be uncomfortable for the participants due to the proximity of the neck muscles. This should be taken into account when determining the intensity of the stimulation. Overall, however, cerebellar TMS is well tolerated and does not induce any specific complaints, apart from nausea [291, 292]. Cerebellar tDCS, on the other hand, does not generate more discomfort than tDCS over other parts of the brain [293].

In the past decade, TMS and tDCS have been used increasingly in cerebellar research [294]. Although the focus was primarily on motor effects and, to a lesser extent, on cognitive effects, some studies have also targeted the social cerebellum. Usually, the vermis, a structure believed to be involved in affective processing [118, 295], is stimulated in these studies. An overview of the studies included in this section is provided in Table 2.

**Table 2:** Overview of cerebellar stimulation studies researching the social cerebellum

Authors	Type of stim	Area of stim	Stim parameters	Social domain	Outcome
Schutter et al. [296]	High-frequency rTMS	Vermis	20 Hz, 15 min, 9000 pulses	Processing of facial expressions	Responsiveness to positive stimuli ↑
Ferrucci et al. [297]	atDCS and ctDCS	Bilateral CB	Reference: R deltoid; 2 mA, 20 min	Processing of facial expressions	Time to identify negative facial expressions ↓
Schutter and van Honk [298]	Low-frequency rTMS	Vermis	1 Hz, 20 min, 1200 pulses	Emotion regulation	Negative mood ↑ after viewing unpleasant pictures
Demirtas-Tatlidede et al. [299]	iTBS	Vermis	20 trains of 10 bursts, 600 pulses	Emotion regulation	No changes in mood
Chen et al. [300]	atDCS; ctDCS	R CB	Reference: R buccinator; 2 mA, 25 min	aMMN and sMMN	atDCS: sMMN ↑, aMMN ~; ctDCS: sMMN ↓, aMMN ~
Lega et al. [301]	Low-frequency rTMS	R CB	1 Hz, 15 min, 900 pulses	Pitch and timbre discrimination	Learning pitch discrimination ↓; no effect on timbre discrimination

*stim* = stimulation; *rTMS* = repetitive transcranial magnetic stimulation; *Hz* = hertz; *min* = minutes; *atDCS* = anodal transcranial direct current stimulation; *ctDCS* = cathodal transcranial direct current stimulation; *CB* = cerebellum; *R* = right; *mA* = milliamperes; *iTBS* = intermittent theta-burst stimulation; *aMMN* = auditory mismatch negativity; *sMMN* = somatosensory mismatch negativity

One aspect of social processing that has been examined is the processing of facial expressions. High-frequency rTMS (20 Hz, 15 min, 9000 pulses) over the vermis increases the emotional responsiveness to positive stimuli (i.e., happy facial expressions), leaving the responsiveness to fearful and neutral expressions unchanged [296]. Bilateral tDCS (anodal and cathodal) significantly reduces the time needed to identify negative facial expressions (anger and sadness) without changing the reaction times to positive (happy) and neutral expressions [297].

Whether cerebellar stimulation can affect the way we react to external emotional stimuli is unclear. Although cerebellar rTMS (1 Hz, 20 min, 1200 pulses) seems to result in an elevated negative mood induced by viewing unpleasant pictures (emotion regulation task; ERT) as compared to sham and occipital stimulation [298], iTBS (20 trains of 10 bursts, 600 pulses) over the vermis does not induce any changes in mood after ERT [299]. This might be the result of a different functional impact of the used stimulation protocols (low-frequency rTMS = inhibitory; iTBS = excitatory).

Early perceptual processing has been shown to relate to social cognition and community functioning in schizophrenic patients [302] and in a healthy population [303]. Early perceptual processing can be easily studied with electroencephalography using event-related potentials such as auditory and somatosensory mismatch negativity (MMN). It has been shown that the cerebellum is involved in the early stages of somatosensory [64] and auditory processing [304]. To the best of our knowledge, only one study examined the impact of cerebellar stimulation on auditory and somatosensory MMN. Chen et al. [300] found that anodal stimulation increased and cathodal stimulation decreased the peak amplitude of the somatosensory MMN, whereas no effect was observed on the auditory MMN (oddball stimuli had a deviant duration in both modalities). However, the authors only looked at the peak amplitude, while Moberget et al. [304] primarily found a difference in peak latency. Therefore, it would be interesting to investigate the impact of cerebellar stimulation on peak latency of the auditory MMN in cerebellar patients as compared to controls. In addition, it has been demonstrated that low-frequency rTMS (1 Hz, 15 min, 900 pulses) over the right cerebellum interferes with learning a pitch discrimination task without effect on a timbre discrimination task [301]. This confirms that the cerebellum is involved in (early) auditory processing in a very specific manner, as also shown by Moberget et al. [304].

The involvement of the cerebellum in social cognition is becoming an accepted notion in the scientific community [20]. Meta-analyses have shown that different areas of the cerebellum can be linked to specific social cognitive processes, such as mentalizing and mirroring [2, 70]. However, studies employing noninvasive brain stimulation and functional imaging data are lacking. Although there is some evidence that cerebellar stimulation might affect emotional processing and early perceptual processing, both involved in adequate social functioning, rigorous studies using cerebellar stimulation are needed to complement the functional imaging and electroencephalogram data. In addition, cerebellar stimulation might be a useful aid in the rehabilitation of social behavior in patients. Early invasive cerebellar stimulation studies by Heath [305] provide some very promising results in a psychiatric population, and experimental animal studies highlight the usefulness of stimulation techniques to improve our understanding of the role of the cerebellum in social behavior [306]. Carta et al. [306], for example, have used optogenetic activation to demonstrate an anatomical pathway between the cerebellum and the ventral tegmental area, a key structure for the processing and encoding of reward. In addition, they showed that activity of this pathway was essential for social preference, which also plays a role in human behavior/society. The development of animal models of social disorders is also crucial to evolve to future clinical applications of noninvasive brain stimulation of the cerebellum, such as a possible rescue of behavioral symptoms related to impaired connectivity between the cerebellum and cerebral cortex as observed in autism [237]. However, factors such as duration of the stimulation, the shape of the electrodes, and montage (location of electrodes) need to be taken into account to optimize electrical/magnetic stimulation over the cerebellum in order to obtain effective cerebellar neuromodulation [307].

### The Effect of Cerebellar Transcranial Magnetic Stimulation on Social Sequencing (Elien Heleven, Frank Van Overwalle)

An increasing number of studies highlight the importance of the cerebellum in social processing, most often in the posterior part (i.e., Crus I and II). To investigate the causal and potentially clinical role of the cerebellum in social functioning, researchers turned to a noninvasive neurostimulation technique: transcranial magnetic stimulation (TMS). TMS is a useful tool to modulate the excitability of a targeted brain region by way of an electric field induced with a pulsed magnetic field using a magnetic coil (for a review, see [294]; see also contribution on “Targeting the social cerebellum by noninvasive neurostimulation” by van Dun and Manto).

We are aware of one study which used TMS to investigate the mediating role of the cerebellum in advanced social functioning without explicit sequencing, more specifically implicit intergroup bias [308]. In this study, participants evaluated trait adjectives that were primed by a picture of an in- or outgroup member, while triple-pulse TMS (20 Hz) was delivered over the right cerebellum between prime and target. Their results revealed that the in-group bias (i.e., faster categorization of positive adjectives when preceded by in-group faces) was abolished by TMS as compared to a pre-TMS baseline and a visual cortex stimulation control group.

In a novel unpublished study, we investigated the effect of TMS on the social cerebellum using the Picture and verbal Story sequencing task developed by Heleven, van Dun, and Van Overwalle [71], where social events (including false and true beliefs) and nonsocial events have to be put in the correct chronological order (see also contribution on “The role of the cerebellum in understanding social sequences” by Heleven and Van Overwalle). This task was administered before and after TMS treatment [309]. We delivered repetitive TMS on the right posterior cerebellum using a double cone coil, at a frequency of 1 Hz, 2 trains of 500 pulses with an intertrain interval of 0.5 s at 80% of the resting motor threshold. Participants were 46 healthy young adults, and half of them received TMS stimulation whereas the other half received a sham treatment. Accuracy rates for the Picture and Story sequencing tests were at ceiling, showing little effects. In contrast, the reaction time data showed a pattern that was quite similar for the Picture and Story sequencing tests, revealing faster responses for the TMS as compared to the sham group after stimulation (*p* = 0.036 and 0.064, respectively). Of most importance, although there was a general learning effect of the sequencing task from pre- to post-stimulus, we observed in both Picture and Story tests a dramatic decrease in response times for the TMS group on all conditions (*p* < 0.001). Conversely, for the sham group, we only observed a significant, but less strong, decrease on only some of the conditions. The general TMS effects on all (i.e., social beliefs and other) conditions suggest that the coil stimulated an extensive part of the cerebellum. Although these results are preliminary, nonspecific, and generally weak, they are promising and seem to suggest a beneficial role on (social) cognitive sequencing of TMS targeted on the cerebellum.

Taken together, research investigating the effects of cerebellar TMS on advanced social functioning is still at its infancy. Although we hypothesize that building internal models of social action sequences is the main role of the posterior cerebellum, brain stimulation can modulate advanced social processes also without explicitly targeting this sequencing function (e.g., [308]). Further research using brain stimulation to modulate the working of the cerebellum in all types of social processing might lead to novel diagnostic tools or clinical treatment methods.

## Discussion and Conclusions

This consensus paper is concluded by highlighting a number of robust findings while pointing out some conflicts and issues where evidence is lacking, along with questions for further research.

### (Frank Van Overwalle)

The aim of this final section is to take stock of the most innovative findings that provided a breakthrough in our thinking on the role of the cerebellum in social cognition and to discuss questions left unanswered that might be addressed in future research.

In the section on mind reading, Maria Leggio put forward the sequencing hypothesis stating that the cerebellum plays a crucial role in identifying sequences in movements and actions which gives humans the social capacity to observe intentions in movements (i.e., mirroring) or attribute mental states in others (i.e., mentalizing), and making predictions about imminent or future social behavior. This sequencing hypothesis was quickly taken up by the team of Van Overwalle and collaborators and put to the test during social mentalizing. They reported robust evidence that processing sequences of actions that allow to infer the state of mind of another person in terms of his or her beliefs (Elien Heleven) or personality traits (Min Pu & Qianying Ma) consistently actives the same area in the posterior cerebellum Crus II with MNI coordinates ± 25 − 75 − 40, in comparison with non-social sequencing. Moreover, dynamic causal modelling studies [23, 24] showed that these cerebellar areas are effectively connected via closed-loops with the bilateral temporo-parietal junction, a key cortical area responsible for taking the mental perspective of another person. This clearly supports the theory that sequencing is an elementary cerebellar process, also for social cognition.

The role of the cerebellum in social cognition is also supported by a novel meta-analysis discussed in the contribution by Qianying Ma and Frank Van Overwalle (“The domain-specific role of the posterior Crus II in social mentalizing”), which points out that the cerebellar Crus II is mainly involved in social mentalizing, and less so Crus I. However, as pointed out by Qianying Ma and Frank Van Overwalle [119], while more anterior Crus II areas (MNI ± 25, − 75, − 40) seem to support sequencing processes in social cognition (and are linked via bidirectional loops to cortical mentalizing areas such as the TPJ; [23, 24]), more posterior Crus II areas (MNI ± 26, − 84, − 32) seem to support social processes without explicit sequencing and are typically activated when reading others’ mental state. This is consistent with the analysis on social cognition in the cerebellum by Xavier Guell, John Gabrieli, and Jeremy Schmahmann (“Relationship between cerebellar social cognition and other motor and non-motor domains”) which attests to the crucial role of the cerebellum in social cognition, but also suggests that this may engage large cerebellar areas without sharp boundaries and localizations. This echoes related claims that lobular boundaries of the cerebellum do not reflect strong functional subdivisions [21, 124]. Given the warning that “lobular divisions have minimal predictive utility” on functional specialization in the cerebellum [34], future empirical studies would benefit from referring to coordinates or subparts of modules, rather than entire lobules.

Taken together, the findings suggest that some areas in the cerebellum might be involved in other social cognitive processes than sequencing. Future research is needed to investigate whether these areas are involved in inferring other mental states (e.g., lower-level intentions, perceived causality, and responsibility) or in other processes underlying social sequencing, such as timing (for instance: in coordinating one’s own or joint actions, in planning interactions ahead), predictions of imminent or future social interactions, or in active inference processes related to social cognition (Marco Michelutti and Arseny Sokolov: “The role of the cerebellum in non-verbal social cognition”). Perhaps the cerebellum might support additional social processes, even including story telling which is typically seen as a language process, although it has clear a social function and sequential event structure. Another interesting avenue for future research is learning social sequences for mentalizing under implicit conditions, that is, without awareness that learning took place (Min Pu, Qianying Ma, and Frank Van Overwalle: “Explicit and implicit learning of social mentalizing sequences“). Although research on implicit learning of mentalizing sequences is almost nonexistent, given that competent social behavior often requires learning of complex implicit rules of social conduct, this research might potentially pave the way for a better understanding and treatment of social processes and impairments from a completely different perspective.

The section on body reading (Marco Michelutti and Arseny Sokolov: “The role of the cerebellum in non-verbal social cognition”) reports that body movements recruit more anterior areas of the posterior lateral cerebellum. Studies on connectivity demonstrate that when observing point-light displays, effective and structural loops are revealed between the anterior Crus I (MNI – 42, − 56, − 32) and the pSTS, a key area in the cortical system for biological movement detection. However, the specialized functional role of various cerebellar areas recruited during motion perception (see Introduction) remains unclear. The issue of location is important, given the general idea that the cerebellum performs a uniform prediction process, with specialization depending on where closed-loops from different cortical areas responsible for motor and nonmotor processes terminate on the cerebellar surface. To illustrate, the functional location and potential role of the anterior Crus I during the perception of body motion (which connects to the pSTS; Marco Michelutti and Arseny Sokolov) seem to be quite different from the posterior Crus II involved in higher-level mentalizing (which connects to the TPJ; [23, 24]). Additional areas, more anterior and inferior in the cerebellum, have been also recruited during action observation (see Introduction, Fig. 1), but their particular functions seem still unclear and perhaps more related to low-level visual processing of movement. Of interest, Chiara Ferrari and Zaira Cattaneo (“TMS and cerebellar regions involved in biological motion perception”) provide in their paper some preliminary evidence that the more anterior medial lobule VI appears to modulate early movement identification, while the more posterior left lobule IV/Crus I is likely to modulate later higher-level cortical processing.

Silvia Clausi, Michela Lupo, and Maria Leggio (“Clinical implications of the role of the cerebellum in mentalizing”) paint in their contribution an overview of the potential clinical implications of the cerebellum on social cognition, and Giusy Olivito, Libera Siciliano, and colleagues (“Connectivity within the cerebello-cerebral mentalizing network and clinical populations”) discuss clinical implication based on an analysis of connectivity in the cerebellar–cerebral circuits. Both contributions support the idea that difficulties in social interactions and personal relationships described in neurodegenerative pathologies and psychiatric populations such as schizophrenia, depression, and bipolar disorder are a direct consequence of brain abnormalities and not a reactive symptom of the neurological diagnosis. Focusing more on a particular patient population, Laura Rice and Catherine Stoodley (“Cerebellar contributions to social behaviors and social networks in autism”) discuss the cerebellar contribution to social behavior in autism. Together, these clinical contributions demonstrate that the paucity of the current empirical data does not allow for a conclusive overview and theory on the specific role of cerebellar–cerebral networks in social deficits in psychiatric and neurodevelopmental disorders. This is just a beginning, and further studies are needed.

Importantly, the current evidence supports the notion that social impairments in several neurodegenerative pathologies are a direct consequence of cerebellar abnormalities, not just side effects. Future research is relevant for a better comprehension of the neurobiological bases of social impairments in these populations, as well as for the implementation of psychological interventions (e.g., psycho-educational treatment, counseling, or cognitive rehabilitation). A more detailed characterization of the cerebellar modulatory function on the cortical processes involved in mentalizing may help to develop specific rehabilitation protocols. Moreover, it will allow clinicians to treat individuals suffering from mentalizing dysfunctions, by implementing behavioral and neurological interventions where the cerebellum is a potential target for (noninvasive) therapeutic intervention to improve social outcomes and behavior.

The final section on cerebellar neurostimulation demonstrates that this is largely an uncharted terrain where scientific progress is badly needed. Kim van Dun and Mario Manto (“Targeting the social cerebellum by noninvasive neurostimulation”) promote the idea that the social cerebellum might be a promising target for noninvasive neurostimulation in various neurodegenerative impairments. However, so far, attempts to improve social understanding by cerebellar stimulation using TMS or tDCS are limited and show ambivalent results (Elien Heleven and Frank Van Overwalle: “The Effect of Cerebellar Transcranial Magnetic Stimulation on Social Sequencing”). Several improvements on current protocols are, however, possible and may involve longer duration of off-line stimulation, or stimulation on-line at the start of the putative social sequencing process. Recent reports on montages that may target social functions and networks in the cerebellum [310] might pave the way for improved cerebellar neurostimulation. However, a limitation of current research is that little is known about other, perhaps behavioral, ways of influencing the cerebellum in addition to noninvasive brain stimulation.

The present consensus paper provides only a short paragraph on animal research in the contribution by Laura Rice and Catherine Stoodley (“Cerebellar contributions to social behaviors and social networks in autism”). This leaves open the question how much the cerebellum contributes to social behaviors in many varieties of animals, especially with respect to higher-order mentalizing capacities which most nonprimate animals seem to lack. However, there are novel ideas for future research that might begin to remedy this omission (see [20]). To illustrate, rats are able to develop prosocial interactive behaviors aimed for the joint solution of complex operant conditioning tasks. For instance, pairs of rats can be trained in adjacent Skinner boxes to obtain a pellet of food when jumping simultaneously on a platform. What is the relationship of such joint behavior with activity in the cerebellum, and how much does this compare to human social understanding and cerebellar functioning?

To conclude, this collection of contributions shows that although the field of the social cerebellum is young, it shows an outburst of novel research findings clearly attesting to the critical functional role of the cerebellum in social cognition and prediction, providing new theoretical insights and new ways for innovative research.
